# Structure-based design of native-like HIV-1 envelope trimers to silence non-neutralizing epitopes and eliminate CD4 binding

**DOI:** 10.1038/s41467-017-01549-6

**Published:** 2017-11-21

**Authors:** Daniel W. Kulp, Jon M. Steichen, Matthias Pauthner, Xiaozhen Hu, Torben Schiffner, Alessia Liguori, Christopher A. Cottrell, Colin Havenar-Daughton, Gabriel Ozorowski, Erik Georgeson, Oleksandr Kalyuzhniy, Jordan R. Willis, Michael Kubitz, Yumiko Adachi, Samantha M. Reiss, Mia Shin, Natalia de Val, Andrew B. Ward, Shane Crotty, Dennis R. Burton, William R. Schief

**Affiliations:** 1 Department of Immunology and Microbiology, The Scripps Research Institute, La Jolla, CA 92037 USA; 2 IAVI Neutralizing Antibody Center, The Scripps Research Institute, La Jolla, CA 92037 USA; 3 Center for HIV/AIDS Vaccine Immunology and Immunogen Discovery, The Scripps Research Institute, La Jolla, CA 92037 USA; 4 Vaccine and Immune Therapy Center, The Wistar Institute, Philadelphia, PA 19104 USA; 5 Department of Integrative Structural and Computational Biology, The Scripps Research Institute, La Jolla, CA 92037 USA; 6 Division of Vaccine Discovery, La Jolla Institute for Allergy and Immunology, La Jolla, CA 92037 USA; 7 Division of Infectious Diseases, Department of Medicine, University of California, San Diego, La Jolla, CA 92037 USA; 8 The Ragon Institute of Massachusetts General Hospital, Massachusetts Institute of Technology and Harvard University, Cambridge, MA 02139 USA

## Abstract

Elicitation of broadly neutralizing antibodies (bnAbs) is a primary HIV vaccine goal. Native-like trimers mimicking virion-associated spikes present nearly all bnAb epitopes and are therefore promising vaccine antigens. However, first generation native-like trimers expose epitopes for non-neutralizing antibodies (non-nAbs), which may hinder bnAb induction. We here employ computational and structure-guided design to develop improved native-like trimers that reduce exposure of non-nAb epitopes in the V3-loop and trimer base, minimize both CD4 reactivity and CD4-induced non-nAb epitope exposure, and increase thermal stability while maintaining bnAb antigenicity. In rabbit immunizations with native-like trimers of the 327c isolate, improved trimers suppress elicitation of V3-directed and tier-1 neutralizing antibodies and induce robust autologous tier-2 neutralization, unlike a first-generation trimer. The improved native-like trimers from diverse HIV isolates, and the design methods, have promise to assist in the development of a HIV vaccine.

## Abstract (web summary)

Eliciting broadly neutralizing antibodies (bnAbs) is a primary HIV vaccine goal, but available immunogens expose epitopes for development of non-nAbs. Here, the authors use computational and structure-guided design to develop improved native-like envelope trimers and analyze Ab response in animal models.

## Introduction

The number of deaths associated with HIV is estimated at 1.1 million people annually, and there are ~2.1 million new infections each year, highlighting the urgent need for an HIV vaccine^1^. During infection, the humoral immune response targets the HIV-1 Env glycoprotein (gp160), and after years of infection, a relatively small fraction of individuals produce potent bnAbs^2^. Passive infusion of bnAbs has been shown to protect against challenge in rhesus macaque and mouse models, suggesting that a bnAb-inducing vaccine could potentially protect humans^3–8^. Many HIV bnAbs are highly mutated, have long variable domain loops, and some exhibit auto- or polyreactivity, features not typically associated with antibodies induced by current licensed vaccines^9–14^. Therefore, induction of bnAbs by vaccination will likely require novel immunization strategies.

Many strategies to obtain broad antibody responses involve vaccination with multiple Envelope proteins in sequence or as a cocktail. Approaches to guide the immune system toward elicitation of a specific bnAb-class such as ‘B-cell lineage vaccine design’^15^ or ‘germline-targeting vaccine design’^11,16–19^, often employ very similar envelope sequences in sequential boosting schemes. Multiple boosts with immunogens containing similar epitopes can produce B-cell competition from alternative B-cell lineages within germinal centers (GCs), as well as due to inter-GC B-cell migration, antibody feedback and memory B cells entering ongoing GCs^20,21^. Immunodominance hierarchies can arise, in which B cells reactive to one epitope expand more than others^22,23^. While the population dynamics of B cells induced by complex antigens is still not completely understood, it is reasonable to hypothesize that reducing the number of competing, undesired epitopes could alter the specificity of the response and favor the development of antibodies against desired epitopes.

The HIV gp160 protein exposes a variety of different B-cell epitopes that induce both nAbs and non-nAbs^24^. Recombinant gp140 proteins stabilized in the prefusion conformation (“native-like” trimers) bind to all but one class of known bnAb and display reduced affinity for several non-nAbs and therefore generally mimic the antigenic profile of the functional membrane-bound gp160^25^. Immunization with stabilized recombinant gp140 variants such as BG505 SOSIP.664 have induced both non-nAbs and nAbs against the autologous virus but have not induced bnAbs in mice, rabbits, guinea pigs or macaques^26–30^. These experiments have revealed three principal targets of non-nAbs on soluble native-like trimers: the V3 loop, the epitopes exposed due to CD4-induced conformational changes (CD4i), and epitopes at the base of the SOSIP trimer artificially exposed due to removal of the transmembrane domain and other portions of gp41^23^. A key goal in this work was to design trimer modifications that would reduce exposure of these prominent non-neutralizing epitopes.

We previously developed two improved variants of BG505 SOSIP.664 with reduced V3 reactivity and increased thermal stability and expression yield by employing cell-surface mammalian display or by introducing an engineered disulfide bond^18^. The trimers, BG505 MD39 and BG505 SOSIP.DS21, respectively, have only been employed as immunogens in a bnAb-germline homozygous knockin mouse model that lacks the ability to mount significant off-target responses^17,18^. Here we sought to further improve on the V3-suppression properties of BG505 MD39 using computational design and confirm suppression of V3-loop immunogenicity of MD39/DS21 variants in rabbit immunizations.

The co-receptor binding site and other non-neutralizing epitopes are buried on the prefusion trimer yet become exposed upon CD4 receptor engagement^31–33^. An intra-protomer disulfide (I201C, A443C) can prevent CD4i conformational changes^34,35^, and mutation of quaternary contacts can reduce CD4 binding by a factor of 20^36^, but neither approach eliminates CD4 binding. HIV Env immunogen binding to CD4+ T cells may have other undesirable immunological consequences, such as potentially reducing the effective dose, masking CD4bs bnAb epitopes, alterating GC reaction dynamics and suppressing immune reponses^37,38^. Therefore, we employed computational design to discover mutations that minimize the affinity for CD4, but otherwise leave the trimer antigenic profile unaffected.

Unlike the membrane-bound HIV Env glycoprotein, soluble native-like trimer immunogens expose a bare protein surface at their base, that is not occluded by the membrane. This surface at the base of the trimer is immunogenic in mice, which is one likely reason why mice produce only non-neutralizing antibodies when immunized with BG505 SOSIP.664^39^. We demonstrate that this exposed surface also induces non-neutralizing antibodies in rabbits and rhesus macaques. We employ structure-guided design to incorporate glycans within and around the base of various native-like trimers to block binding of base-directed antibodies.

## Results

### Native-like trimers with reduced V3 reactivity

**Fig. 1:**
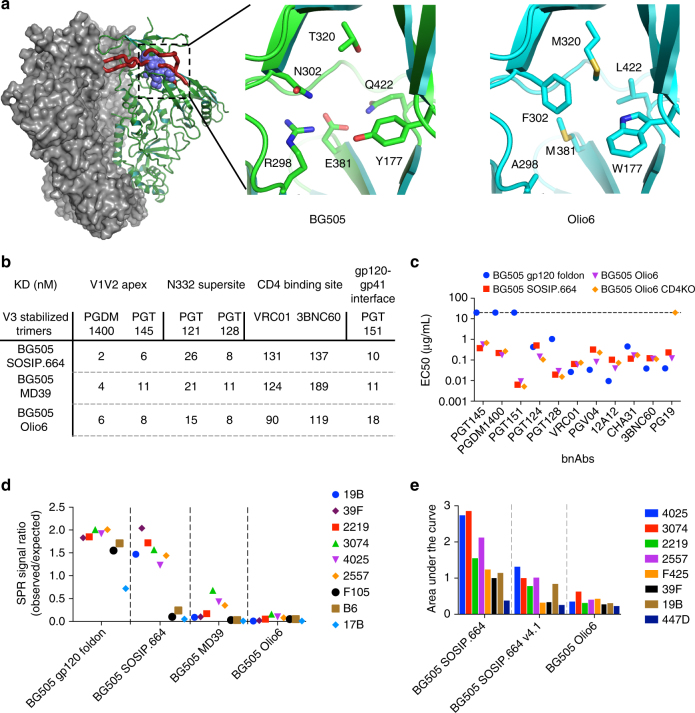
Structure-based design of BG505 Olio6 trimer. **a** Structural model of BG505 SOSIP.664 trimer (PDBID: 4TVP), with two subunits shown as a grey surface, one subunit shown as green trace. The V3 loop is shown as a thick red trace and the six substituted positions are shown as slate spheres. The enlarged frames show the six substituted positions in sticks, in green are the BG505 amino acids and in cyan are the corresponding Olio6 amino acids. **b** Binding affinities of bnAbs as measured by SPR are shown for BG505 SOSIP.664, BG505 MD39, and BG505 Olio6. The *K*_D_s for V1V2 apex bnAbs were measured with trimer analyte and IgG ligand, but for other bnAbs Fab analyte and trimer ligand were used. **c** bnAb EC_50_ values were measured by ELISA for stabilized trimers. When an EC_50_ could not be determined, the symbol was placed at the highest concentration tested (20 μg/mL). **d** Binding of non-nAbs to trimers measured by SPR. Trimers were analytes at 1 μM. The SPR signal ratio is the observed response normalized to the expected response of one gp120 per antigen-binding site. **e** Binding of V3 non-nAbs to trimers by ELISA. BG505 SOSIP.664 v4.1 is reported in de Taeye et al. 2015 ^26^. The Area Under the Curve has units of Abs 450 nm×log(dilution). Measurements are done in duplicate and represent two independent experiments

To develop BG505 MD39 variants with further reduced V3 exposure, we sought to engineer mutations that stabilize the native-like conformation and limit the conformational dynamics that expose non-neutralizing epitopes. We devised a structure-based computational design strategy and employed the Rosetta Design platform to carry out this strategy^40^. We hypothesized that the network of buried hydrophilic amino acids (Y177, R298, N302, T320, E381, Q422) near the base of the V3 loop could support a conformation with an exposed V3 loop and nearby contacting regions. Thus, we employed Rosetta Design simulations to replace buried hydrophilic residues with hydrophobic ones at the base of the V3 loop and the underlying gp120 core, in order to energetically stabilize the conformation of the V3 loop seen in the crystal structure (Fig. 1a).

We expressed and purified three designs, subjected them to biophysical evaluation, and found that one was best for silencing V3 non-nAb reactivity. This design, BG505 Olio6, has six mutations relative to MD39 at buried positions: Y177W, R298A, N302F, T320M, E381M, Q422L. According to design models, the redesigned residues in BG505 Olio6 have improved hydrophobic packing, with only 1 buried polar atom and 1 unsatisfied hydrogen bond, compared to 11 buried polar atoms and 6 unsatisfied hydrogen bonds among the same residues in BG505 SOSIP.664 or MD39. BG505 Olio6 forms homogenous trimers as assessed by SEC-MALS, has a melting temperature of 76 °C (Supplementary Fig. 1 and Supplementary Table 1), and retains a native-like trimer antigenic profile, with high affinity for bnAbs according to SPR and ELISA (Fig. 1b, c). To assess V3 loop exposure, we employed SPR to evaluate binding to a panel of four V3-directed non-nAbs for which high-resolution structures of Fabs bound to V3-peptides are available (2219, 3074, 4025, and 2557). BG505 Olio6 shows a reduction in binding to V3-directed antibodies relative to a non-native trimer with a fully exposed V3 epitope (BG505-gp120-foldon) and to the native-like trimer BG505 SOSIP.664 (Fig. 1d). We also measured binding to a panel of eight V3 antibodies by ELISA, the results of which demonstrated that BG505 Olio6 has lower V3 reactivity than BG505 SOSIP.664 and BG505 SOSIP.664.v4.1^26^ (Fig. 1e). In a related study, NHP immunizations with BG505 Olio6 further validated the V3-suppression described here^30^. Thus, computational redesign of buried polar regions underneath the V3 loop of an HIV-1 Env trimer has produced an improved V3-lockdown, native-like trimer immunogen.

### Native-like trimers with altered CD4 binding properties

**Fig. 2:**
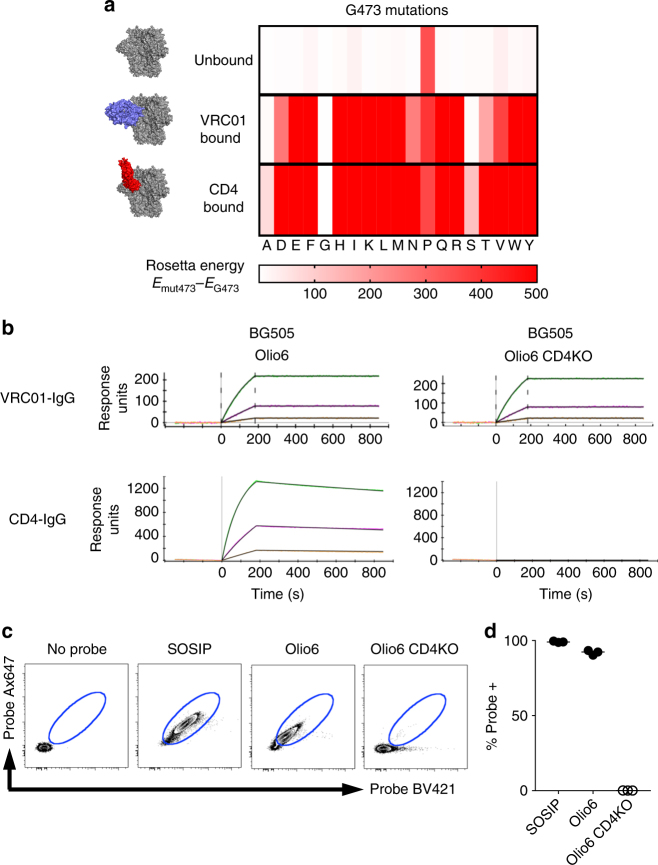
Design and evaluation of the CD4-KO mutation. **a** The difference in computed Rosetta energy between trimers with glycine at position 473 and all 20 amino acids except cysteine at position 473 in three states: unbound, VRC01-bound, and CD4-bound. Energies are colored from white to red, with energies similar to glycine in white and unfavorable energies in red. **b** SPR binding of trimers to VRC01-IgG and CD4-IgG. IgGs were ligands and trimers were analytes. **c** Binding of different trimers to CD4^+^ T cells. Fluorescently labeled Env trimer probes were mixed with human PBMC. All panels gated on CD4^+^ T cells. Blue oval gate indicated probe positive population. A ‘no probe’ staining was used as a negative control to set the lower bound of the positive gate. **d** Frequency of Env trimer probe positive CD4^+^ T cells. *N* = 3 individual human donor samples for each probe. The data are representative of two experiments with a total of four independent samples

To inhibit CD4 receptor engagement, we sought to find mutations in the CD4-binding site of HIV-1 Env^41,42^ that prevent CD4 binding without hindering the binding of CD4bs-directed bnAbs, such as VRC01-class bnAbs, and without adversely impacting the overall native-like antigenic profile of MD39 or Olio6. We considered three configurations: the unliganded HIV trimer, the HIV trimer bound with bnAb VRC01, and the HIV trimer bound with CD4. We sought to discover sequences that favor the unliganded and VRC01-bound states but disfavor the CD4-bound state. To solve this multistate design problem, we employed a computational saturation mutagenesis approach that mutates each position in the CD4bs to all 20 amino acids except cysteine for each of the three states (see Methods section). To identify mutations that produce an energy gap between the VRC01-bound and CD4-bound states, we evaluated the energy of each mutation. One mutation, G473T, had the desired energy profile: no change in energy for the mutation in the unliganded state, minimal change in energy in the VRC01-bound state, and large unfavorable energy change in the CD4-bound state (Fig. 2a). In support of this finding, it has been shown that an alanine at 473 in gp120 reduces CD4 binding and enhances b12 binding^43^. The BG505 Olio6 trimer containing the G473T mutation (referred to as BG505 Olio6.CD4KO) retained an overall antigenic profile consistent with a stabilized native-like trimer (Fig. 1c). To assess CD4 reactivity, we tested binding of BG505 Olio6.CD4KO to CD4-IgG. In an SPR experiment in which CD4-IgG was captured on the sensor and trimers were analytes at concentrations of 1000, 250, and 52.5 nM, binding to BG505 Olio6 was detected at all concentrations, but binding to BG505 Olio6.CD4KO was not detected at any concentration (Fig. 2b). In the same experiment, we tested binding to seven different VRC01-class bnAbs. All VRC01-class antibodies bound to BG505 Olio6.CD4KO, and 6 out of 7 VRC01-class bnAbs bound with nearly identical kinetics to trimers with and without the CD4KO mutation (Fig. 2b and Supplementary Fig. 1). We found similar results by ELISA (Fig. 1c), suggesting the CD4KO trimer still has high affinity for VRC01-class bnAbs. Two other mutations (G473A, G473S) were identified by our multistate design simulation that also showed reductions in CD4 binding (Fig. 2a and Supplementary Fig. 1E).

We further assessed whether BG505 Olio6.CD4KO could interact with membrane-bound CD4 receptors on human CD4^+^ T cells. Human peripheral blood mononuclear cells (PBMC) were interrogated by flow cytometry using fluorescently-labelled native-like Env trimers. BG505 SOSIP.664 and Olio6 bound to 99% and 93% of CD4^+^ T cells, respectively, whereas BG505 Olio6.CD4KO trimer bound to only 0.1% of CD4^+^ T cells (Fig. 2c, d), a ~1000-fold reduction in binding. Thus, computational multistate design produced a native-like trimer with significantly reduced CD4 binding and largely unaltered VRC01-class and other bnAb antigenicity and minimal non-nAb reactivity (Fig. 1c and Supplementary Fig. 1).

### Cleavage-independent trimers via circular permutation

**Fig. 3:**
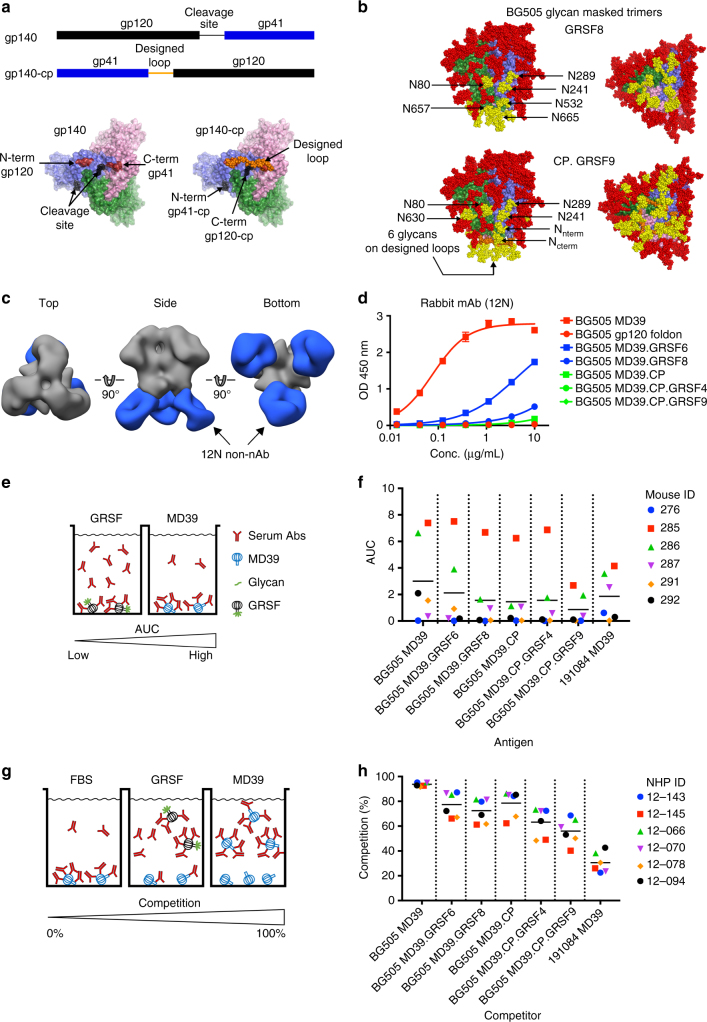
Circular permutation and glycan-masking of BG505 MD39 trimer. **a** gp140 trimer gene layout for standard native-like trimers (labeled gp140) and for circular permuted trimers (labeled gp140-cp). The trimer is shown in surface representation, subunits are colored green, blue and light pink. A model of the designed loop is shown in orange spheres. **b** A structural model of BG505 MD39 GRSF6. The trimer is colored as in **a**. Native glycans are shown in red spheres, and designed glycans are shown in yellow spheres. Each designed glycan is labeled in HXB2 numbering **c** Negative-stain EM 3D reconstruction of the base-binding monoclonal 12N Fab (blue) in complex with BG505 SOSIPv4.1 (grey). **d** The ELISA data for monoclonal rabbit antibody, 12N, binding to trimer variants. **e** ELISA assay format for testing VRC01 gH mouse sera. **f** ELISA AUC data for sera from six VRC01 gH mice binding to various trimers. ELISA antigens labeled on the *x*-axis and mouse identifiers in the legend. The Area Under the Curve has units of absorbance×log(dilution). **g** ELISA assay format for testing rhesus macaque sera. **h** The ELISA AUC data for sera from six BG505 Olio6-immunized rhesus macaques binding to various trimers. ELISA antigen is BG505 MD39, the competitors are listed on the *x*-axis, and the monkey identifiers are given in the legend. All trimers were made in 293F cells, unless otherwise noted. Measurements are done in duplicate and represent two independent experiments

Various nucleic acid-based (NA) technologies have emerged as promising vaccine platforms for inducing humoral immunity^44–47^, yet it is unclear if stabilized native-like HIV trimers will be amenable to NA technology, since protease cleavage and extensive purification are generally required to obtain native-like trimers. Gp140 trimers encode a furin cleavage site between gp120 and gp41. One strategy to remove the cleavage dependence on native-like trimers is to replace the furin cleavage site with a flexible linker^48–50^. In this strategy, the linker connects the C-terminus of gp120, at the base of the trimer, to the N-terminus of gp41. The linker therefore might impede elicitation of neutralizing antibodies to the C-terminus of gp120 or to part of gp41. We devised a new strategy for generating cleavage-independent trimers by swapping the order of gp41 and gp120 in the gene (i.e., circular permutation or CP) (Fig. 3a). This CP strategy also requires a designed linker, but in this case the linker does not block potential neutralization epitopes because it only covers the bottom of the trimer that is not exposed on membrane-bound spikes. This alternative linker location also offers improved opportunities for glycan masking of the base, as described below. The resulting trimer, BG505 MD39.CP, does not require furin cleavage and has a native-like antigenic profile except for reduced PGT151 binding owing to trimming of the gp41 N-terminus in the design. Also, the V3-reactivity is reduced compared to BG505 SOSIP.664 but increased relative to BG505 MD39 (Supplementary Fig. 2).

### Native-like trimers with masking of the base

In order to focus immune responses to bnAb epitopes at membrane-distal regions of the trimer (CD4bs, V2 apex and N332 supersite epitopes), we aimed to glycan mask three exposed surfaces near the base of the BG505 SOSIP.664 trimer structure (PDB id: 4TVP). The first under-glycosylated region on BG505 is at positions 241 and 289, which are highly conserved PNGS among HIV isolates and have been previously identified as a major target of strain-specific nAbs in BG505 SOSIP.664-immunized rabbits^51^. A second under-glycosylated area is close to the N-terminus of gp120 and at the subunit interface, near position 80. Although the surface patch around N80 is somewhat conserved, we added a glycan at position 80 because it is not part of the CD4bs, V2 apex, or N332 supersite epitopes on which our vaccine efforts are focused. A third under-glycosylated area is the bottom of the trimer near the C-terminus of gp41 (positions 630–664) that is occluded by the membrane in functional Env spikes. We report two new BG505 MD39-based trimers with glycan-masking at these sites: BG505 MD39 GRSF6 with five extra PNGS at N80, N241, N289, N653 and N665, and BG505 MD39 GRSF8 with six extra PNGS at N80, N241, N289, N532, N657, and N665 (Fig. 3b). We also report two new glycan-masked BG505 MD39.CP-based trimers: BG505 MD39.CP.GRSF4 with PNGS at N80, N241, N289, and two in the linker, and BG505 MD39.CP.GRSF9 with PNGS at N80, N241, N289, N630, two in the linker, and one each at the N- and C-termini (Fig. 3b). The GRSF trimer variants have antigenic profiles consistent with a native-like trimer, except for reduced PGT151 binding by CP trimers and reduced 35O22 binding by GRSF8 and CP.GRSF9 (Supplementary Fig. 2).

To determine if glycan masking could block a gp41-dependent, non-neutralizing antibody elicited from a rabbit, we assessed binding to the mAb 12N isolated from a rabbit immunized with BG505 SOSIP.664^51^. We obtained a low resolution negative-stain reconstruction of 12N binding to BG505 SOSIP v4.1 showing the non-nAb bound to the bottom of the trimer (Fig. 3c). The 12N mAb was tested by ELISA for binding to various trimers. BG505 MD39 without any glycan masking bound strongly to 12N, while BG505 MD39.GRSF6, and BG505 MD39.GRSF8 had reduced binding, and the CP trimers (BG505 MD39.CP, BG505 MD39.CP.GRSF4, and BG505 MD39.CP.GRSF9) showed no appreciable reactivity (Fig. 3d). Thus, rabbits elicit non-neutralizing antibodies directed to the bottom of a soluble native-like trimer, and at least one epitope targeted by rabbits can be masked by GRSF trimers.

To further test the glycan masking of the GRSF trimers, we assessed reactivity of mice immunized with BG505 SOSIP trimers. In a previous study, we immunized germline heavy-chain VRC01 knockin mice (VRC01-gH mice) with germline-targeting and boosting immunogens^19^. The immunized mice developed a VRC01-like antibody response, as reported, but also developed off-target responses, as 15% of the B cells in these mice express normal mouse heavy and light chains^52^. One group of mice received the VRC01 germline-targeting prime immunogen eOD-GT8 60mer^53^, followed by three BG505 SOSIP-based trimer immunogens. Each of the BG505 SOSIP.664-based trimers contained the three exposed patches described above. Sera from six mice in this group were tested by ELISA for reactivity to BG505 MD39 and various GRSF variants (Fig. 3e). Each of the trimers used as ELISA antigens included the N276 glycan in the VRC01 epitope, which in this case, prevented binding by VRC01-class antibodies and allowed binding only by off-target antibodies. BG505 MD39 reacted with four of the six sera, the GRSF trimers generally reduced binding to all sera, and BG505 MD39.CP.GRSF9 showed little or no binding to any of the sera (Fig. 3f and Supplementary Fig. 3). Therefore, most of the immunogenic B-cell epitopes other than the VRC01-class epitope in this mouse model were blocked by glycan masking the bottom of the trimer and the glycan holes on the lower parts of the trimer.

To further examine the effects of glycan masking, we assessed reactivity to sera from six rhesus macaques immunized three times (week 0, 8, and 24) with the BG505 Olio6 trimer. The sera neutralized BG505 pseudovirus and induced the lowest V3-specific non-nAb mean responses in that study^30^. Here we were interested in the ability of GRSF-based trimers to reduce reactivity to the exposed surface patches described above. The sera contain antibodies against many epitopes, and therefore we designed a competition ELISA assay in which we depleted the sera with different trimer variants and then detected residual antibody binding to BG505 MD39 trimer coated on the ELISA plate. This allowed us to directly compare the impact of glycan-masking in different constructs (Fig. 3g). We computed a percent competition to quantitate the amount of antibody response that is absorbed by the competing trimer (see Methods section). When using a matched BG505 MD39 trimer as both the competitor and the coating antigen on the ELISA plate, almost complete competition (mean 94%) was observed. A trimer from an unrelated strain, 191084, was used as a competitor to determine a minimal level of cross-reactivity, and we found that 191084 absorbed only 31% of the reactivity induced by BG505. The glycan resurfaced trimers, BG505 MD39.GRSF6 and BG505 MD39.GRSF8, reduced binding of the serum to BG505 MD39 coated on the ELISA plate by 77% and 72%, respectively. The CP trimer with no extra glycans reduced binding by 79%, yet adding glycans to exposed surfaces led to lower competition of 63% and 56% for BG505 MD39.CP.GRSF4 and BG505 MD39.CP.GRSF9, respectively (Fig. 3h and Supplementary Fig. 3B). These findings suggest that the three exposed surface patches are immunogenic in macaques immunized with the native-like BG505 Olio6 trimer, and that antibodies to these epitopes can be blocked by GRSF trimers.

### Transfer of stabilizing mutations to alternate strains

Broadly neutralizing antibodies can accommodate significant epitope diversity found among HIV isolates, and therefore it has been suggested that immunization regimes focused on induction of bnAbs could include cocktails of native-like HIV trimers^11,17–19,54^. However, native-like trimers have been produced for only a limited number of strains, and in the experiments reported thus far, immunogenicity can vary quite considerably^55^. Many HIV Env sequences with SOSIP.664 modifications do not produce native-like trimers of sufficient yield and quality for practical use^55^.

**Fig. 4:**
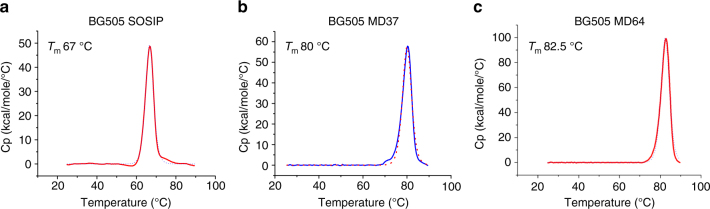
Thermostability of BG505 variants SOSIP, MD37 and MD64. **a** Differential Scanning Calorimetry (DSC) profile for BG505 SOSIP, the raw data are shown as a solid line and fit is shown as a dashed line. Melting Temperature (*T*_m_) values from the fit are shown. **b** DSC profile for BG505 MD37 **c** DSC profile for BG505 MD64. The data are from one experiment

To construct native-like trimers from new strains, we designed four sets of stabilizing mutations. Two variants (MD39 and Olio6) have been described (refs. ^18^ and above) and in addition, we designed two new variants (MD37 and MD64), each with improved thermal stability over MD39. Key mutations in MD64 were obtained by screening a BG505 SOSIP.664 whole gene saturation mutagenesis library for improved antigenic profile trimers (see Methods section). We identified two mutations (A73E and S110E) that increase the melting temperature of BG505 SOSIP.664 by 10 °C and then incorporated these into MD39, which required reversion of three MD39 mutations (E106T, H570V, and D568L). MD64 has a melting temperature of 82.5 °C, an improvement over BG505 SOSIP.664 by 16.5 °C and BG505 MD39 by 5.5 °C (Fig. 4 and Supplementary Table 1). The second variant, MD37, included many mutations from MD39 and two extra disulfide bonds to stabilize the V3 loop (V120C, Q315C) and to prevent CD4i conformational changes (I201C, A433C), the former of which was described in ref.^18^ and the latter described in refs.^34,35^. BG505 MD37 has a melting temperature of 80 °C (Fig. 4, Supplementary Table 1). MD37 and MD64 also suppress non-nAb binding similarly as MD39 (Supplementary Fig. 4).

**Fig. 5:**
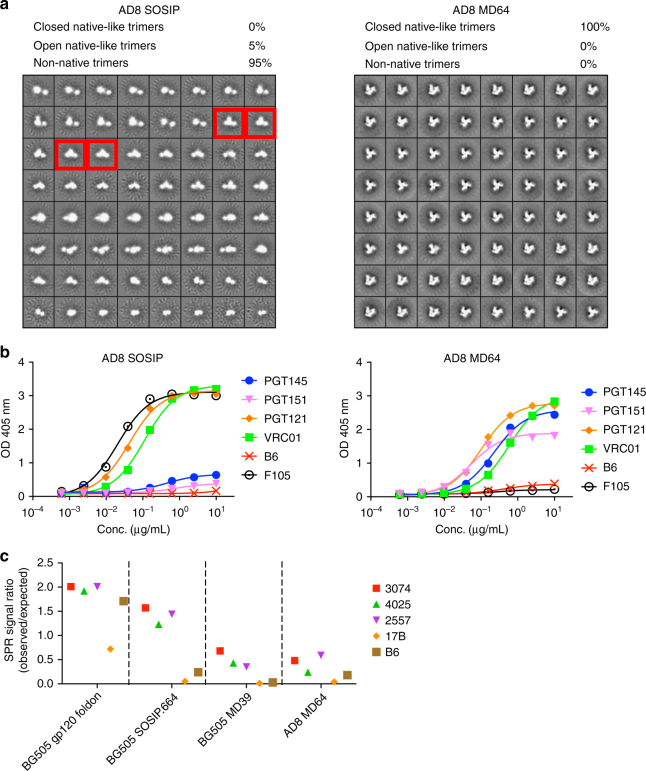
Structural and antigenic assessment of AD8 trimer with different stabilization designs. **a** 2D class averages from negative-stain EM analysis of AD8 SOSIP trimer and AD8 MD64 trimer, classified as closed native-like, open native-like, or non-native. Red boxes indicate examples of open native-like trimers. **b** ELISA-binding curves for bnAbs and non-nAbs against AD8 SOSIP and the AD8 MD64 trimer. ELISA antigens are labeled on top of the plots, and the antibodies are labeled in the legend. **c** SPR signal ratio for non-nAbs binding to AD8 MD64 trimers. The data are from one experiment and measurements are done in duplicate

**Fig. 6:**
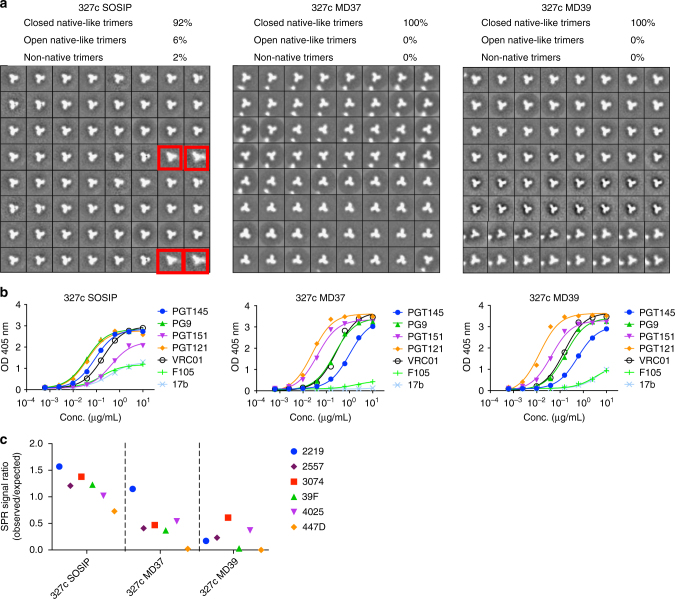
Structural and antigenic assessment of 327c trimer with different stabilization designs. **a** 2D class averages from negative-stain EM analysis of 327c SOSIP, 327c MD37 and 327c MD39 trimers, which are classified as closed-native-like, open native-like or non-native. Red boxes indicate examples of open native-like trimers. **b** ELISA-binding curves for bnAbs and non-nAbs against 327c SOSIP, 327c MD37 and 327c MD39 trimers **c** SPR signal ratio for the V3 non-nAbs binding to 327c SOSIP, 327c MD37 and 327c MD39 trimers. The data are from one experiment and measurements are done in duplicate

Our goal was to create trimers from multiple different clades that could be of potential utility for SHIV challenge studies and for sequential boosting studies of VRC01-class responses using isolates with diverse CD4bs properties. We chose two clade B strains (AD8 and SF162P3) and one clade C strain (327c) that have well-characterized SHIV challenge stocks available^56,57^, thus enabling potential protection studies in NHPs immunized with matched native-like trimers. Viral isolates potently neutralized by bnAbs are of interest in studies aiming to re-elicit those bnAbs^58^. For use in immunization strategies aimed at re-elicitation of VRC01-class antibodies, we constructed native-like trimers of 191084 (clade A), 001428 (clade C) and AC10 (clade C). The six trimers display an antigenic profile similar to BG505 stabilized native-like trimers (Supplementary Table 1). In some cases, the mutations described here were required to produce well-formed native-like trimers. For example, negative-stain EM imaging of AD8 SOSIP showed that 95% of the trimers were non-native (poorly folded) and the remaining 5% were open (flexible) native-like trimers. In contrast, AD8 MD64 showed 0% non-native trimers and 100% closed (well-folded) native-like trimers (Fig. 5a). AD8 SOSIP binds the non-nAb F105 and does not bind well to trimer-specific bnAbs. AD8 MD64, however, has minimal binding to non-nAbs and binds well to trimer-specific bnAbs (Fig. 5b, c). While the SOSIP mutations were sufficient to form native-like 327c trimers, both the 327c MD37 and 327c MD39 variants showed improved native-like trimer formation, as assessed by negative-stain EM (Fig. 6a). The additional mutations did not affect bnAb binding, except for APEX bnAb affinity reductions by factors of 2.0 and 2.6 for 327c MD37 and 327c MD39, respectively (Fig. 6b and Supplementary Table 1), and provided reduced V3 reactivity, similar to BG505 variants harboring those mutations (Fig. 6c). 327c MD37 had an increased melting temperature of 4–8 °C over the other 327c variants (Supplementary Table 1). We note that not all mutation sets are compatible with all isolates. The full sequences of the 327c, AD8, and BG505 modified trimers can be found in Supplementary Fig. 6. Thus, we identified four different sets of stabilizing mutations in BG505 and demonstrated that Env from other isolates can often be stabilized by transfer of at least one of these mutation sets.

### In vivo properties of stabilized 327c native-like trimers

We investigated the immunogenicity of three 327c native-like trimers in rabbits: 327c SOSIP.664, MD37 and MD39. Three groups of rabbits (*n* = 5) were immunized with 50 µg of either 327c SOSIP.664, MD39 or MD37 protein. Iscomatrix was used as a strong adjuvant for all immunizations. Rabbits were immunized at weeks 0, 8, 24, and 32, following a similar immunization scheme that was recently shown to increase the induction of Tier 2 nAb titers in rhesus macaques^30^. Intra-muscular immunizations were given bilaterally into the hind-legs, meant to increase systemic recruitment of circulating antigen-specific B and T cells into GCs, thus improving nAb development^59^.

**Fig. 7:**
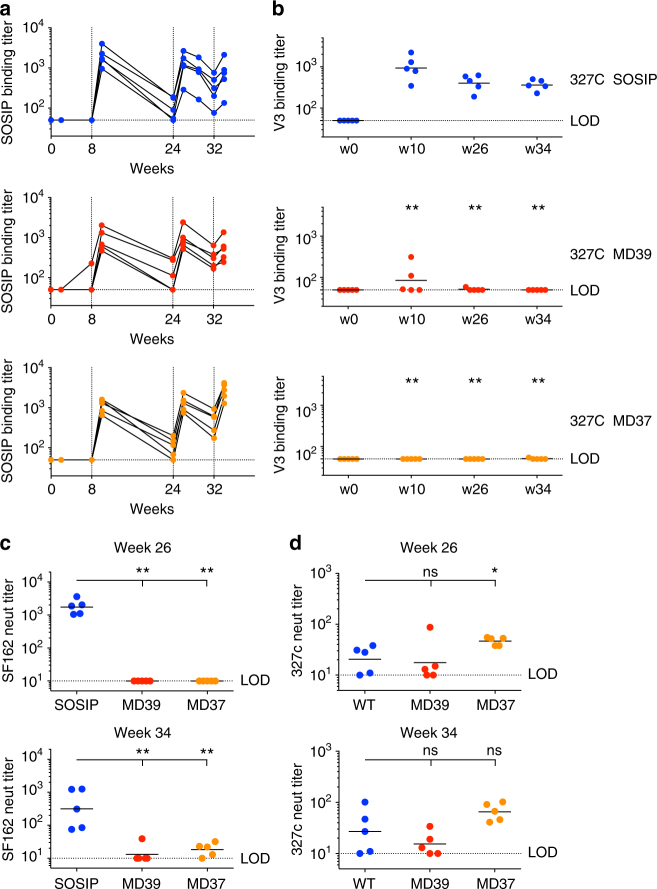
Rabbit immunization with 327c native-like trimers. **a** 327c trimer binding titers at week 0, week 8, week 10, week 24, week 26, week 32, and week 34 time points for rabbits immunized with 327c SOSIP, 327c MD39, and 327c MD37. **b** V3 binding titers at week 0, week 10, week 26, and week 34 time points for rabbits immunized with 327c SOSIP, 327c MD39, and 327c MD37. The V3 peptides correspond to the V3 peptide sequence found in the matching trimer immunogen. Stars indicate the significance of differences between WT and respective stabilized designs. **c** Tier-1 SF162 neutralization titers at week 26 and week 34. **d** Autologous 327c Tier-2 neutralization titers at week 26 and week 34. Group size *N* = 5. The data are from one experiment. Statistical comparisons were calculated using two-tailed Mann–Whitney *U* tests. * indicates *p* < 0.05, ** indicates *p* < 0.01

We observed strong trimer EC_50_ ELISA-binding titers for all immunogens following the second, third and fourth immunizations (Fig. 7a). Binding titers slowly declined between immunizations, and peaked at week 26, following the third immunization in 327c SOSIP.664 and 327c MD39 immunized animals. Animals immunized with 327c MD37 developed the highest peak titers in the study, following the fourth immunization (1:1200–1:4000). To evaluate the reduction in V3-directed Abs, we measured V3 loop peptide-binding ELISA titers against the respective WT, MD39 and MD37 peptides (Fig. 7b). The V3 loop EC_50_ binding titers in 327c MD39 and 327c MD37 immunized groups were undetectable or sharply reduced compared to the SOSIP group, across all time points (*p* < 0.01, Mann–Whitney *U* test). To ensure that induced Abs could cross-react between WT and engineered V3 loop peptides, we tested sera from 327c SOSIP.664-immunized animals on WT, MD39 and MD37 V3 loop peptides, and we found no significant differences in binding titers (Supplementary Fig. 5A). To assess the ability of stabilized trimer designs to suppress V3-directed non-nAb induction, we tested neutralization against isolates SF162 (Fig. 7c) and MW965 (Supplementary Fig. 5B) in TZM-bl cell neutralization assays. Native-like trimer-induced neutralization against tier-1 viruses, such as SF162 and MW965, is driven by multiple non-nAb specificities, most notably V3-directed and, to a lesser degree, CD4i-directed Abs^28^. Whereas SOSIP-immunized animals mounted substantial SF162 nAb titers after three or four immunizations, MD39- and MD37-immunized animals had no detectable SF162 neutralization following the third immunization (*p* < 0.01, Mann–Whitney *U* test), and significantly reduced titers following the fourth immunization (*p* < 0.01, Mann–Whitney *U* test). Lastly, we investigated the induction of autologous and heterologous tier-2 nAb titers in all groups. Most immunized animals developed robust nAb titers against the 327c strain following the third and fourth immunizations (Fig. 7d). MD37 immunization elicited the highest geometric mean nAb titers of the three groups after three (week 26) or four (week 34) immunizations, and the MD37-induced titers were significantly higher than SOSIP-induced titers at week 26, but there were no significant differences in 327c nAb titers among the three groups at wk34 (Mann–Whitney *U* test, Fig. 7d). Furthermore, MD37-immunized animals had a 100% response rate, whereas the MD39 and SOSIP groups had lower response rates. All sera were tested for neutralization on a panel of 12 heterologous tier-2 viruses representing global HIV diversity^60^, and no tier-2 cross-neutralization was detected (Supplementary Fig. 5C). Taken together, the results show that both 327c MD39 and MD37 effectively suppressed the induction of V3-directed non-nAbs, and 327c MD37 elicited the most robust and consistent autologous nAb responses in vivo.

## Discussion

Recombinant HIV trimer immunogens tested in standard animal models have thus far elicited non-nAbs as well as nAbs against both the autologous isolate and Tier-1 isolates, but elicitation of heterologous Tier-2 nAbs has been elusive. New immunogens that focus responses away from non-neutralizing or isolate-specific epitopes and toward relatively conserved epitopes may be required to induce bnAbs. Here we employed computational and structure-guided protein design to develop trimers with reduced reactivity to several classes of non-nAbs. This included a modification that reduces reactivity to CD4 that should reduce exposure of CD4-induced non-neutralizing epitopes as well as reduce the potential for trimer sequestration on and activation of CD4^+^ T cells, and reduce masking of CD4-binding site epitopes targeted by bnAbs in human vaccination. We show that the design interventions impact the specificity of induced antibody responses to native-like trimers. The modifications are transferable to other isolates and therefore could be utilized in other native-like trimer immunization strategies to reduce undesired immune responses.

The structural integrity of vaccine antigens exposed to conditions and factors in vivo may have profound effects on available B-cell epitopes and the resulting antibody response. Detecting immune response differences due to changes in structure is challenging because we may not understand and we may lack the ability to reproduce in vitro, the variety of non-native conformations that potentially can be induced in vivo. Thus, we may only detect a subset of such changes in structure and the associated undesired antibody responses. Most soluble native-like trimers reported to date either undergo conformational breathing, which exposes the V3 loop or include a subpopulation of non-native-like trimers that expose the V3 loop in vivo^26,28,61^. By comparing complexes of V3-peptides with non-nAbs (2219, 3074, 4025 and 2557) to structures of unliganded native-like trimers, it is apparent that binding of such non-nAbs to a trimer requires a V3 loop conformational change to expose the epitope, and this must be accompanied by a concomitant exposure of protein surfaces that normally contact the V3 loop such as the V2 loop and a patch consisting of the top of the α1 helix, the ß3 strand and the ß21 strand. It is reasonable to assume that a portion of the antibody response to most soluble native-like trimers also targets these newly exposed regions as well as the V3 loop. Therefore, design interventions that can help retain structural integrity in vivo, such as those reported here, may help reduce elicitation of non-neutralizing responses to native-like trimer immunogens.

Due to relatively high sequence conservation of the CD4-binding site residues among HIV-1 isolates, the CD4KO mutation described here should be widely applicable to any CD4bs-containing, Envelope-based immunogen. The strong (1000-fold) reduction in CD4KO trimer binding to human CD4+ T cells in vitro suggests that similar effects may be obtained in vivo. Because the CD4KO mutation is a single amino acid change and is nearly agnostic to bnAb reactivity, it would be reasonable to consider including the mutation in native-like trimer immunogens tested in human clinical trials.

The truncation of gp160 at position 664 to create soluble gp140 immunogens is widely used in the Env design field. However, the artificial proteinaceous “bottom” of the molecule is a large off-target epitope capable of generating non-nAb responses^39^. Here we showed that glycan masking at strategic positions on the trimer can block some of the antibody responses generated from a multi-boost immunization protocol with native-like trimers in mice, rabbits and macaques. Overall, the glycan masked construct most effective at blocking bottom-directed Abs was BG505 MD39.CPGRSF9, in which the circular permutation positioned a linker favorably for bottom masking. It remains to be determined whether immunizations with bottom-glycan-masked trimers will elicit lower levels of bottom-directed responses and potentially re-direct the immune response toward less immunogenic Tier-2 epitopes. Additional off-target antibody responses might engage the variable loops or surface patches on the bottom that may still be accessible due to incomplete glycosylation. Mapping of off-target responses will be important to guide engineering of additional silencing modifications.

The SOSIP modifications (SOS disulfide and I559P mutation) have been a critical advance for stabilizing Env trimers into a pre-fusion state, but the SOSIP modifications alone do not yield well-behaved trimer immunogens for all HIV-1 isolates. Here, the MD64 modifications enabled formation of well-folded AD8 native-like trimers, whereas the SOSIP modifications alone did not. AD8–SHIV infection of rhesus macaques is the only reported case of potent HIV bnAb development in non-human primates^62^. Our development of an AD8 native-like trimer (AD8-MD64) now enables a study of autologous challenge after native-like trimer vaccination in non-human primates.

Glycan holes, higher thermal stability, and multivalent antigen presentation have all been suggested as factors leading to higher autologous antibody titers. Env trimers from a majority of HIV-1 isolates are missing at least one >50% conserved glycoslyation site, and the hole(s) created by the missing glycan(s) can be targeted by autologous neutralizing antibodies^51^. The 327c isolate and our 327c trimer immunogens, however, have all highly conserved glycans intact, which may have limited the nAb titers in our rabbit study. The elimination of V3-directed immunodominant epitopes was not sufficient to cause large increases in autologous neutralization titers, although the MD37-induced autologous nAb titers were significantly higher than SOSIP-induced titers after three immunizations and were more consistent as well. However, since the 327c study aimed to isolate the effects of V3 reduction, the bottom of the trimer was not resurfaced and therefore likely generated a competing non-nAb response that may have impeded development of stronger nAb responses. In related findings from a recent NHP study comparing four different trimer platforms (BG505 SOSIP, BG505 SOSIP v5.2 (more advanced version of v4.1), BG505 SOSIP.NFL, and BG505 SOSIP.Olio6), Olio6, with or without the CD4KO mutation, elicited the lowest V3 ELISA responses and tier-1 V3-directed neutralization but did not elicit higher autologous BG505 neutralization^30^, however the data in Fig. 3h shows that bottom-directed responses contributed substantially to the overall response. In our rabbit study, the highest and most consistent autologous titers were induced by the most thermally stable immunogen in the experiment, 327c-MD37. More studies will be needed to understand the relationship between thermal stability and neutralization titer^27^. Beyond thermal stability, multivalent display of glycoprotein immunogens using liposomes^63^ or self-assembling nanoparticles^64,65^ may facilitate increased autologous and heterologous titers.

The design principles reported here for HIV-1 trimers potentially can be widely used for engineering vaccines against other pathogens. Suppression of undesired conformational dynamics by computational stabilization of buried polar networks, such as in the Olio6 design, should be applicable to other glycoprotein vaccine targets with known structures (e.g., influenza hemagglutinin or respiratory syncytial virus F-protein). The multistate design algorithm for knocking out binding to cell-surface receptors while preserving binding of neutralizing antibodies targeting the same site could be employed for a wide range of pathogens.

In summary, by using computational and structure-guided design, we have developed a series of modifications that improve the antigenicity and stability of native-like HIV trimers. We have demonstrated that the modified trimers can mask undesired epitopes and can alter immune responses in a predictable way. Integrating the advances reported here with other vaccine concepts, such as germline targeting, may help focus the antibody response toward bnAb epitopes, and thus toward the development of an HIV vaccine.

## Methods

### Computational design of HIV Env

The Rosetta Design computational simulations used the 4TVP^42^ trimer structure and identified positions to mutate as the buried hydrophilic positions near the base of the non-neutralizing V3 loop epitope. Nearby positions (8 Å) were allowed to sample multiple conformations but not change amino acid identity. For the buried positions, we restricted the amino acids to a set of hydrophobic amino acids. Combinations of mutations were selected that were tightly packed, as assessed by RosettaHoles^66^, and formed low energy configurations filtered for those with favorable values of structure-derived energy terms (Lennard-Jones, number of buried unsatisfied hydrogen bonds and total Rosetta energy). BG505 Olio6 was one of the lowest energy designs that were assessed as recombinant proteins. In a second series of design simulations, a multistate model was used to discern mutations in the CD4-binding site that did not affect the binding energy of VRC01-class antibodies, but significantly reduced binding energy of soluble CD4. The crystal structures 4TVP (BG505 SOSIP trimer unbound), 3NGB (VRC01 bound^67^,) and 3JWO (CD4 bound^41^,) were used as input to the simulations. The sequence of the BG505 strain was modeled on to the bound-state structures and relaxed using Rosetta. Next, we ran a computational saturated mutagenesis protocol, evaluating the energy of each amino acid except Cys at every position proximal to the CD4-binding site in the context of all three structures. The mutations with the biggest energy difference between CD4 bound and VRC01 bound were selected. The resulting design discussed in this manuscript was BG505 Olio6 CD4KO. In a third series of design simulations using the designLinearSegments program of MSL^68^, we circular permuted BG505 SOSIP.MD39 by searching for loops in the protein structure database of various lengths to connect the C-terminus of gp41 to the N-terminus of gp120. We also searched for loops that could connect residues near the ends of gp120 and gp41 (up to 5 residues from the termini). We manually curated the set of potential loops by assessing the number of similar length loops found in natural structures and the geometry of the connections between the new loop and terminal residues. The selected CP design had the C-terminus of gp41 trimmed back to residue 660 to direct the polypeptide chain in the direction of the N-terminus of gp120, which was connected at residue 34 and was made of glycine and serine amino acids to avoid clashes (Supplementary Fig. 7). The Rosetta code is available via https://rosettacommons.org. The MSL code is available via http://msl-libraries.org.

### Design of native-like trimers by mammalian display

With the exception of the disulfide bonds, mutations in MD64 and MD37 that were not present in MD39 were identified by screening a BG505 SOSIP.664 whole gene saturation mutagenesis library by mammalian cell surface display for improved antigenic profile (i.e. PGT145 and PGT151 high/B6 and 4025 low) as described previously^18^.

### 327C and AD8 SOSIP.664 design

SHIV-327c (327C) and SHIV-AD8 (AD8) SOSIPs were designed similarly as for BG505 SOSIP.664. In brief, 327C and AD8 *Env* genes were synthesized codon-optimized (Life Technologies—GeneArt) bearing the SOS and IP mutations, and were truncated at amino acid 664. The cleavage sites were modified to ‘RRRRRR’ to increase furin cleavage. Synthesized 327C SOSIP.664 and AD8 SOSIP.664 constructs were sub-cloned into the pcDNA3.1 expression vector and the sequence was confirmed by Sanger-sequencing.

### Gene synthesis and protein production

The genes encoding the native-like trimers and HIV bnAbs were synthesized by GenScript. The antibodies and trimers were expressed in FreeStyle 293F cells (Invitrogen, Cat no. R79007), which are derived from a low-passage Master Cell Bank and certified mycoplasma free (low-passage numbers from frozen aliquots are used to prevent mycoplasma contamination and maintain cell viability). The constructs were purified in two steps by affinity chromatography using a GE HisTrap column and size-exclusion chromatography using a GE S200 Increase column as described previously^18^. For the cleavage-independent samples, furin protease was not added. The proteins were all aliquoted at 1 mg/mL and rapidly frozen in thin-walled PCR tubes prior to use. The molecular weight and the homogeneity of the trimers reported here were confirmed by size-exclusion chromatography-multi-angle light scattering (SEC-MALS) in PBS using a GE S200 Increase column operating with an isocratic flow of 0.5 mL/min followed by DAWN HELEOS II and Optilab T-rEX detectors (Wyatt Technology). The protein conjugated method was run to assess the portion of mass attributed to the protein from the mass of the glycans (ASTRA). Antibodies were purified by a Capture Select IgG-CH1 Column (ThermoFisher Scientific, Cat. No. 494320005) according to manufacturer’s instructions.

### Antigenic profile characterization of native-like trimers

Capture Enzyme-linked immuno-sorbent assays (ELISAs) were performed both to analyze bNAbs and non-nAbs binding to native-like trimers and to assess conformational/structural integrity and functionality of glycan-masked trimers. Capture antibodies were coated onto the ELISA plates (Corning™ 96-Well Half-Area Plates, Catalog #3690) O/N at 4 °C at 2 or 8 μg/mL in 25 μL PBS pH 7.4 (Thermo Scientific, Catalog #10010-023) per well for assays detecting monoclonal and polyclonal antibodies, respectively, and incubated ON at 4 °C. Human PGT121 Fab was used for trimers and non-nAbs capture (see Gene Synthesis and Protein Production), rabbit His-tag Antibody (GenScript, Catalog #A00174-40) was used for bnAbs capture. Plates were washed 3× with PBS containing 0.2% (v/v) tween (PBST) (Tween 20, Sigma Catalog #P1379-1L) in an 405 TS Washer (BioTek Instrument) and blocked with PBST containing 5% (w/v) skim milk (BD Difco™ Skim Milk Catalog #232100) and 1% (v/v) Fetal Bovine Serum (FBS) (Thermo Fisher, Catalog #16000044) for 1 h at RT. Plates were then washed 3× and 25 μL of dilution series of primary Abs (see Gene Synthesis and Protein Production) in PBST + 1% FSB were added for 1 h at 37 °C. Different starting concentrations for mAbs were determined used with 3-fold or 5-fold dilution series. Plates were washed 5× and mAbs binding was detected adding 25 μL of Anti-Human IgG, Fcγ fragment specific, (Jackson ImmunoResearch Catalog #109-035-098). Both HRP-conjugated secondary antibodies were added at 1:5000 dilution in PBST + 1% FBS. After 1 h incubation at RT, plates were washed 3× and 25 μL TMB Chromogen Solution (Thermo Fisher Catalog #002023) substrate was added. To stop the reaction, 25 μL 0.5 M H_2_SO_4_ were added after 10 min for end-point titers. Absorption was read at 450 and 570 nm on a VERSA max plate reader (Molecular Devices, USA). Background subtraction was performed by subtracting the 570 nm value from the corresponding 450 nm value. The data were subsequently analyzed in Prism (Prism v7; GraphPad Software, La Jolla, USA), using specific equations for dose–response curves to show binding traces and to calculate the 50% effective concentrations (EC50). EC50 were defined as the concentration at which half the maximum signal was reached. For Area Under Curve (AUC), Prism computes it by the trapezoidal method, which is based on connection of a straight line between every set of adjacent points defining the curve, and on a sum of the areas beneath these areas

### Serum ELISA of VRC01-gH mice and rhesus macaques

Macaque sera was obtained from a separate study^30^. As reported in that manuscript, the animals were outbred Indian rhesus macaques (Macaca mulatta) sourced and housed at Alphagenesis, Yemasee, SC and maintained in accordance with NIH guidelines, and the studies were approved by the appropriate Institutional Animal Care and Use Committees (IACUC). VRC01-gH sera was obtained from a separate study^19^. Serial dilutions of macaque sera in dilution buffer (PBS + 0.02% [v/v] Tween20 + 1% [v/v] fetal bovine serum) were incubated in presence of 100 µg/mL of various competitors overnight at 4 °C. ELISA plates were coated with BG505 MD39 overnight at 0.5 µg/mL in PBS, blocked with 5% (w/v) milk in dilution buffer and serum/competitor mixtures were added for 1 h at 37 °C. Bound antibodies were labeled with peroxidase-conjugated anti-human IgG (Jackson ImmunoResearch), plates were developed with TMB Chromogen Solution (Thermo Scientific) and absorption was read on a VersaMax plate reader (Molecular Devices). After background subtraction, areas under the curve (AUCs) were calculated and the data were normalized using the formula Competition = (1—AUC (Competitor) / AUC (FBS only control)) × 100%. The data were plotted in Graphpad Prism. The VRC01-gH mouse sera was run the same without a competitor.

### Negative-stain electron microscopy

12N IgG^51^ was produced in HEK-293F cells, purified by ProteinA affinity chromatography (GE Healthcare), and dialyzed into Tris-buffered saline (TBS). 12N IgG was digested into Fab by incubating with 25% w/w papain resin (Thermo Scientific) overnight at 37 °C and purified using a ProteinA spin column (Thermo Scientific). Six fold molar excess 12N Fab was incubated with BG505 SOSIP.v4.1^26^ at room temperature overnight. A total of 3 μL of purified trimer or 12N Fab + BG505 SOSIP v4.1 complex was adsorbed onto glow discharged carbon-coated Cu400 EM grids and blotted after 10 s. The grids were then stained with either 3 μL of Nano-W (Nanoprobes, Inc.) for 7 s, blotted, and stained again for 15 s followed by a final blot or 3 μL of 1% uranyl formate for 20 s, blotted, and stained again for 1 min followed by a final blot. Image collection and data processing was performed as described previously^26^ on either an FEI Tecnai T12 microscope (2.05 Å/pixel; 52,000× magnification) or FEI Talos microscope (1.98 Å/pixel; 72,000× magnification). 2D classification, 3D sorting and refinement of the 12N Fab + BG505 SOSIP complex was conducted using Relion 1.4^69^. 2D class averages representing top and bottom views of trimers were classified as representing either a closed native-like state, open native-like state, or non-native state^26^. Side views were classified as either closed native-like or non-native.

### Human CD4^+^ T cell Env trimer binding assay

Flow cytometry was used to assess Env trimer binding to human CD4^+^ T cells. Peripheral blood (LRS leukoreduction tubes) from healthy donors were purchased frozen from the San Diego Blood Bank. Frozen human PBMC were thawed and stained with fluorescently labeled Env trimer probes for 30 min. For each Env trimer construct, two fluorescently labeled probes were independently generated using biotinylated trimers conjugated to either streptavidin (SA) linked Alexa Fluor 647 (Invitrogen) or SA linked Brilliant Violet 421 (BD Biosciences). Next a mixture of antibodies [CD4 BV650 (BioLegend, 317436, 1:100), CD20 BV570 (BioLegend, 302332, 1:100), CD16 APCe780 (Thermofisher, 47-0168-42, 1:100), CD14 APCe780 (Thermofisher, 47-0149-42, 1:100), and APCe780 Live-dead stain (Thermofisher, 65-0865-14, 1:1000)] was added to the cells to discriminate cell populations. The cells were washed twice, fixed in 1% (v/v) formaldehyde, and acquired on a BD Fortessa flow cytometer. Flow cytometry data was analyzed in FlowJo.

### Rabbit immunizations and serum preparation

Female New Zealand White rabbits 4 months of age were immunized intramuscularly and bilaterally into the hind legs. All immunizations contained a total of 50 µg of protein, adjuvanted with 75 IscoUnits of ISCOMATRIX split equally between the two sites. Serum blood was collected in vacutainer tubes (BD), incubated for 30 min at room temperature (RT) and clotted blood was spun down at 2000×*g* for 10 min at RT to isolate serum. Serum was heat-inactivated at 56 °C for 1 h and subsequently sterile filtered using 0.22 µm Spin-X tubes (Corning) at max. speed in a table centrifuge, until all serum passed the filter. In determining the number of animals used in our experiments we refer to “Statistical Aspects of Planning and Design of Immunological Experiments“^70^. In measurements of relevant parameters of B-cell function (including Ab production), the standard deviation approximates the mean, in order to detect a two-fold difference between experimental groups, a group size of 5–7 animals is needed. To minimize animal use, we opted for 5 animals per experimental group. No randomization method was used in the study to select animals. The investigator was not blinded to the group allocations. No animals were excluded from the study. All procedures were carried out under strict ethical guidelines and protocols approved by The Scripps Research Institute IACUC committee.

### Pseudovirus neutralization assays

Replication incompetent HIV pseudovirus was produced by co-transfecting *Env* plasmids with an *Env*-deficient backbone plasmid (pSG3∆*Env*, AIDS Reagents Program, Cat# 11051) in HEK293T (ATCC Cat# CRL-11268) cells in a 1:2 ratio, using the X-tremeGENE 9 transfection reagent (Roche). Pseudovirus was collected after 48–72 h by sterile filtration (0.22 µm) of cell culture supernatants, and neutralization was assessed by incubating pseudovirus and serum for 1 h at 37 °C before transferring onto TZM-bl cells as previously described^30^. Neutralization was measured in duplicate wells within each experiment. All nAb titers for group comparisons were measured in two or more independent experiments that were subsequently averaged. Neutralization was tested starting at 1:10 serum dilution followed by nine serial 3-fold dilutions to ensure the highest sensitivity and range of detection. Neutralization IC_50_ titers were calculated using the ‘One site—Fit logIC50’ regression in Graphpad Prism v7.0. IC_50_ nAb titers of incomplete neutralization curves that reached at least 50%, but <90% maximal neutralization, were calculated by constraining the regression fit through 0% and 100% neutralization, to ensure accurate calculation of half-way (50%) nAb titers. All neutralization titers are reported as IC_50_ values. Statistical comparisons between groups of 5 animals were calculated using non-parametric, unpaired, two-sided Mann–Whitney *U* tests. No estimate of variation within groups was used.

327c neutralization was tested using a pseudo-typed version of the SHIV-327c molecular clone^56^. In brief, SHIV-327c *Env*, comprised of the human Tier 2, Clade C CA327 *Env* gene truncated at position 773 and fused to the KB9 transmembrane domain (terminal 98 aa), was cloned into the pSVIII vector using the ‘*Env* A’ and ‘*Env* M’ primers, as previously described^71^. Integrity and sequence of the sub-cloned SHIV-327c *Env* gene was confirmed by Sanger sequencing. Heterologous neutralization breadth was tested on a panel of 12 cross-clade isolates, reported to be most representative of larger virus panels comprised of global isolates from diverse clades^60^.

### Serum ELISA and antigenic profile of 327c/AD8 trimers

Microlon 96-well plates (Corning) were coated overnight with mouse anti-His antibody at 2.5 μg/mL (Thermo Scientific) in phosphate-buffered saline (PBS) at 50 μL per well. Plates were then washed 4–5 times with PBS-Tween (0.05% v/v) and blocked with PBS + 3% (w/v) BSA for 1 h at room temperature. His-tagged 327c SOSIP.664, 327c MD39, 327c MD37, AD8 SOSIP.664 or AD8 MD64 protein was added at 1 μg/mL in PBS + 1% (w/v) BSA for 2 h at room temperature. Plates were then washed 4–5 times with PBS-tween (0.05%) and serially diluted sera or mAbs in PBS + 1% BSA were then added for 1 h at room temperature. Plates were then washed 4–5 times with PBS-tween (0.05%) and alkaline phosphatase-conjugated goat anti-rabbit IgG (Jackson ImmunoResearch) for rabbit sera, or goat anti-human IgG (Jackson ImmunoResearch) for human mAbs, was added for 1 h at a 1:1000 dilution (final concentration 0.33 μg/mL) in PBS + 1% BSA at room temperature. Plates were then washed 4–5 times with PBS-tween (0.05%) and absorption at 405 nm was measured following addition of phosphatase substrate in alkaline phosphatase buffer. EC_50_ binding titers were calculated using Graphpad Prism v7.0. V3-peptide ELISA assays were performed exactly as SOSIP.664 ELISAs, with the following modifications. Microlon 96-well plates (Corning) were coated overnight with 327C V3-peptides (WT IRPNNNTRKSIRIGPGQTFFAT or MD39 IRPNNNTVKSIRIGPGQTFFYT or MD37 IRPNNNTRKSIRIGPGCTFFAT) at 2.5 μg/mL in PBS. Negative and positive control antigens were included (BG505-gp120-foldon or BG505 SOSIP) to validate the antibodies.

### Data availability

The authors declare that the data supporting the findings of this study are available within the article and its Supplementary Information files, or are available from the authors upon reasonable request.

## Electronic supplementary material

Supplementary Information
