# Supplementary material for: Structure-based design of native-like HIV-1 envelope trimers to silence non-neutralizing epitopes and eliminate CD4 binding
Source: Nat Commun. 2017 Nov 21;8:1655. doi: 10.1038/s41467-017-01549-6 (PMC5698488; doi:10.1038/s41467-017-01549-6)
Supplement: Supplementary file 1 — Supplementary Information [file 41467_2017_1549_MOESM1_ESM.pdf]

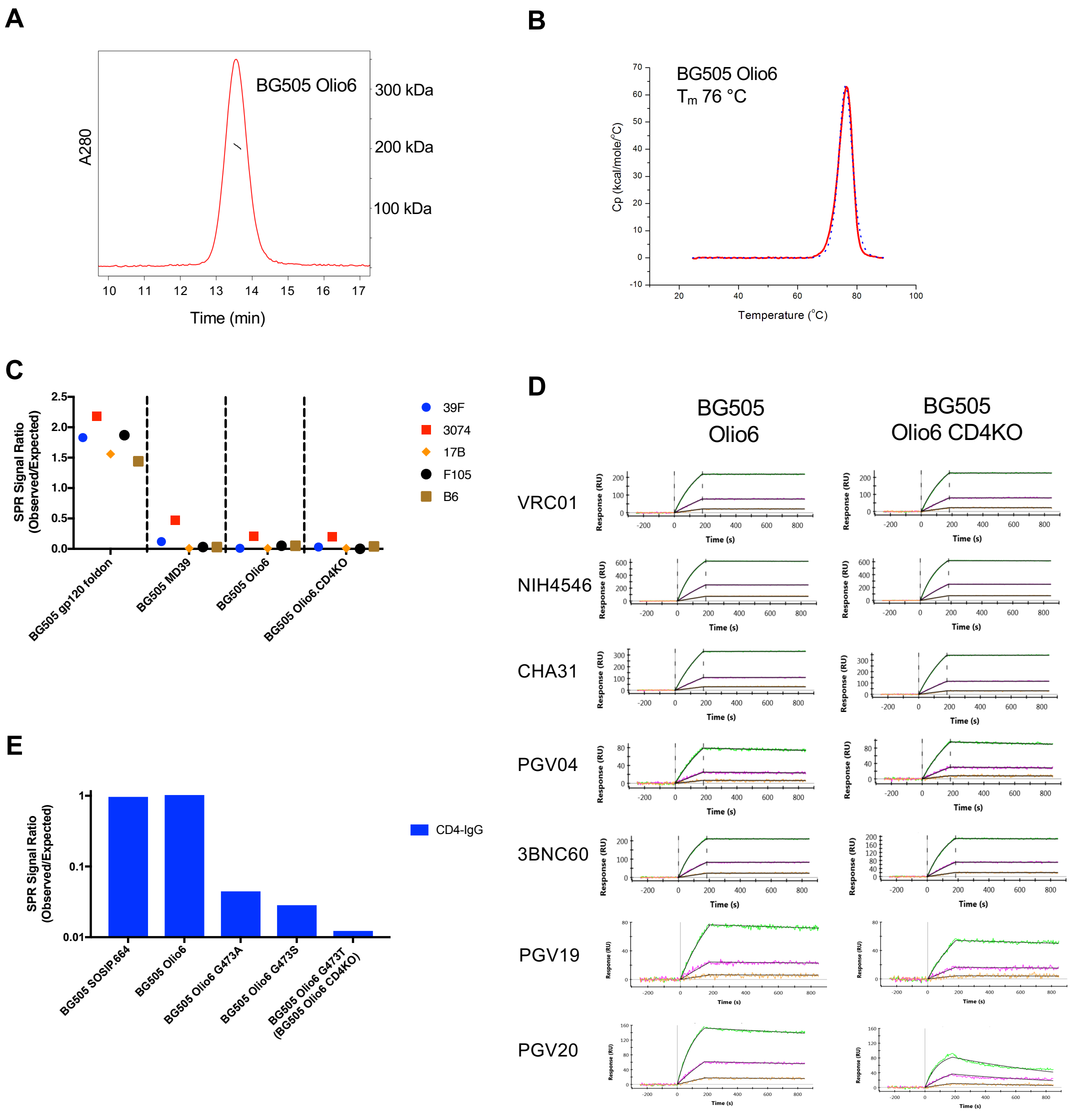

Supplementary Figure 1. Biophysical characterization of BG505 Olio6, BG505 MD64 and VRC01-class bnAbs binding to BG505 Olio6 CD4KO. (A) SEC-MALS trace for BG505 Olio6, with A280 on the left y-axis and protein mass assessed by the protein-conjugate method shown as a solid line inside the peak corresponding to the right y-axis. (B) DSC trace for BG505 Olio6. (C) SPR signal ratio for the non-nAbs binding to BG505 Olio6 CD4KO. BG505 gp120 foldon, BG505 MD39 and BG505 Olio6 were run as controls. (D) The SPR sensograms are shown for VRC01, NIH4546, CHA31, PGV04, 3BNC60, PGV19 and PGV20. IgGs were captured on the SPR chip and trimers (BG505 Olio6 and BG505 Olio6 CD4KO) were flowed as analytes. (E) SPR signal ratio for CD4-IgG captured on the SPR chip and trimers flowed as analytes. Measurements are done in duplicate and represent two independent experiments. Data in panel E was from a single experiment.

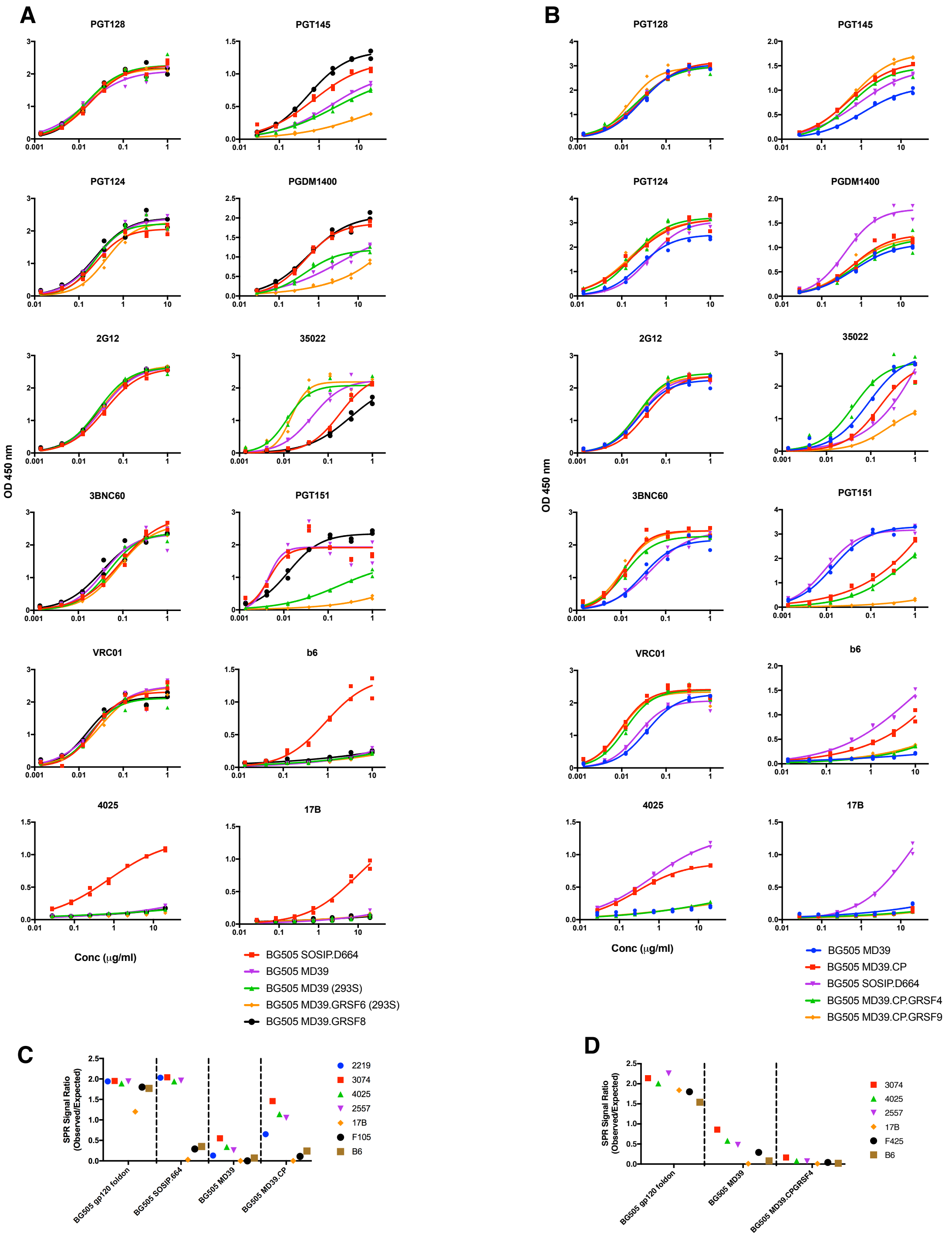

Supplementary Figure 2. Antigenic profile for CP and GRSF trimers. (A) ELISA binding curves for bnAbs and non-nAbs against BG505 MD39.GRSF6, BG505 MD39.GRSF8 (B) ELISA binding curves for bnAbs and non-nAbs against BG505 MD39.CP, BG505 MD39.CPGRSF4, BG505 MD39.CPGRSF9. (C) and (D) SPR Signal Ratio for CP and CP.GRSF4 trimers. BG505 gp120 foldon, BG505 SOSIP.664, BG505 MD39 were run as controls. Data are from one or more experiments and measurements are done in duplicate.

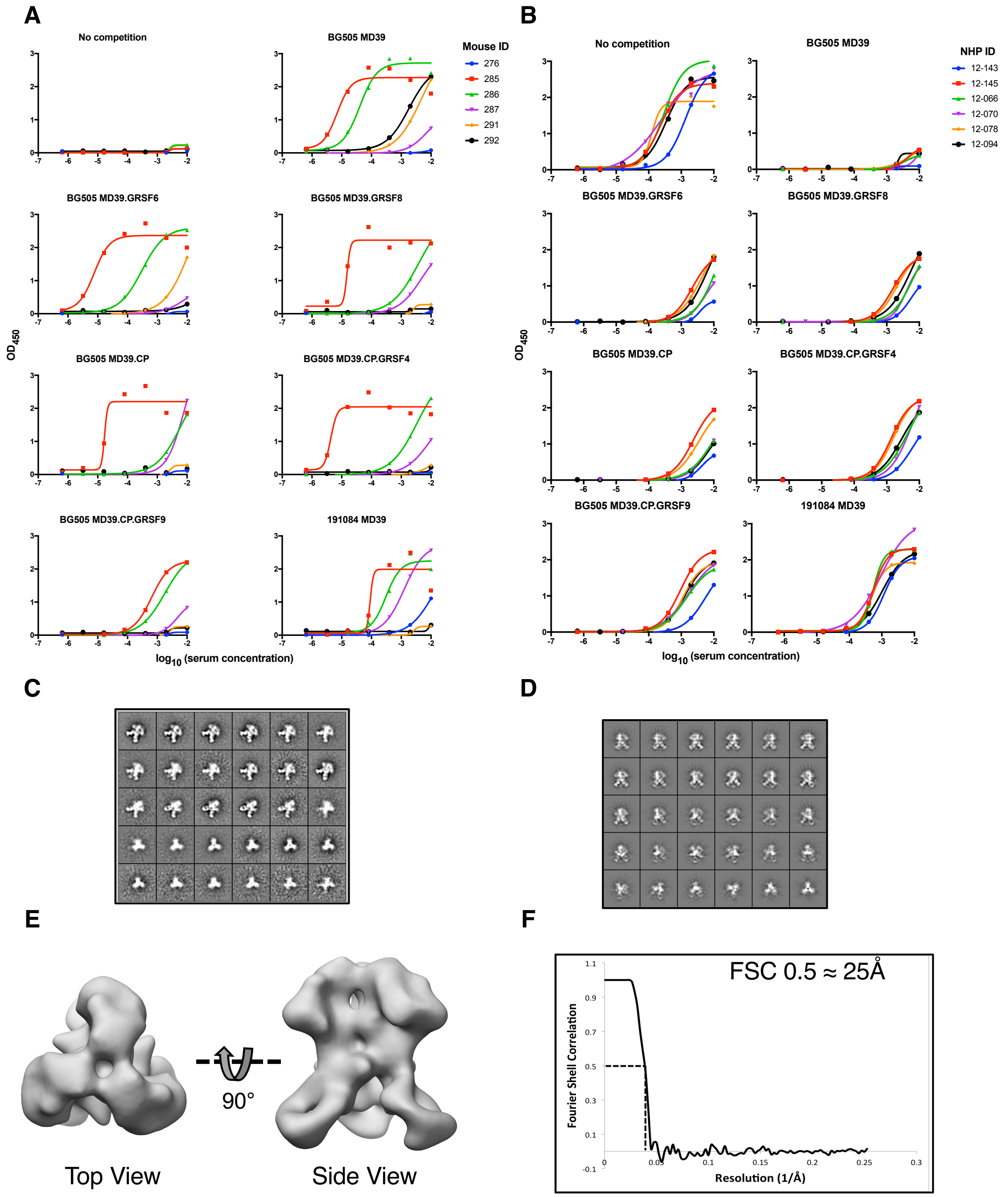

Supplementary Figure 3. Binding and structural assessment of NHP, mouse and rabbit antibodies. (A) ELISA curves of mouse sera binding to trimers used to compute AUC values in Figure 3D (B) ELISA curves of NHP sera binding to trimers used to compute % competition values in Figure 3F (C) Negative-stain reconstruction of rabbit mAbs 12N binding to BG505 SOSIP v4.1 trimer. Reference-free 2D class averages from negative-stain EM analysis of 12N Fab + BG505 SOSIP v4.1 complex. (D) 2D back-projection images of derived from the 12N Fab + BG505 SOSIP v4.1 complex 3D reconstruction. (E) 25Å 3D reconstruction of 12N Fab + BG505 SOSIP v4.1. (F) Fourier Shell Correlation (FSC) curve with estimated resolution using an FSC cut-off of 0.5. Measurements are done in duplicate and represent two independent experiments.

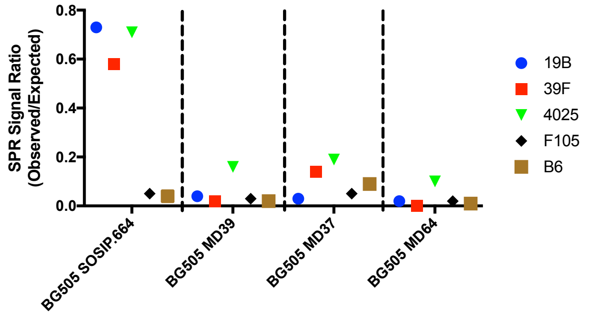

Supplementary Figure 4. Binding of non-nAbs to BG505 MD37 and BG505 MD64. SPR Signal Ratio for BG505 MD37 and BG505 MD64 as measured on a Biacore 4000 instrument. BG505 SOSIP.664 and BG505 MD39 were run as controls. Data are from one experiment.

A

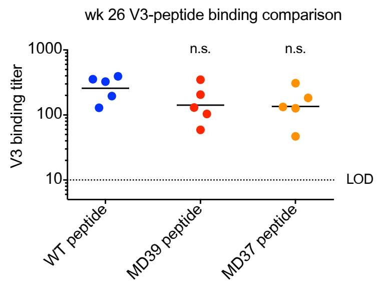

B

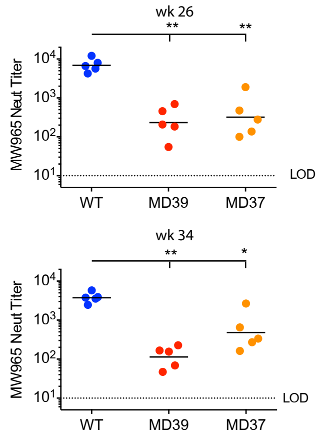

C

		Immunogen: 327c WT				
	Clade	**r8317**	**r8318**	**r8319**	**r8320**	**r8321**
398F1	A	< 50	< 50	< 50	< 50	< 50
246F3	AC	< 50	< 50	< 50	< 50	< 50
CNE55	AE	< 50	< 50	< 50	< 50	< 50
CNE8	AE	< 50	< 50	< 50	< 50	< 50
X2278	B	< 50	< 50	< 50	< 50	< 50
TRO11	B	< 50	< 50	< 50	< 50	< 50
BJOX2000	BC	< 50	< 50	< 50	< 50	< 50
CH119	BC	< 50	< 50	< 50	< 50	< 50
CE0217	C	< 50	< 50	< 50	< 50	< 50
CE1176	C	< 50	< 50	< 50	< 50	< 50
25710	C	< 50	< 50	< 50	< 50	< 50
X1632	G	< 50	< 50	< 50	< 50	< 50

		Immunogen: 327c MD37				
	Clade	**r9720**	**r9721**	**r9722**	**r9723**	**r9724**
398F1	A	NT	NT	NT	NT	NT
246F3	AC	< 50	< 50	< 50	< 50	< 50
CNE55	AE	< 50	< 50	< 50	< 50	< 50
CNE8	AE	< 50	< 50	< 50	< 50	< 50
X2278	B	< 50	< 50	< 50	< 50	< 50
TRO11	B	< 50	< 50	< 50	< 50	< 50
BJOX2000	BC	< 50	< 50	< 50	< 50	< 50
CH119	BC	< 50	< 50	< 50	< 50	< 50
CE0217	C	< 50	< 50	< 50	< 50	< 50
CE1176	C	< 50	< 50	< 50	< 50	< 50
25710	C	< 50	< 50	< 50	< 50	< 50
X1632	G	< 50	< 50	< 50	< 50	< 50

		Immunogen: 327c MD39				
	Clade	**r9085**	**r9086**	**r9087**	**r9088**	**r9089**
398F1	A	< 50	< 50	< 50	< 50	< 50
246F3	AC	< 50	< 50	< 50	< 50	< 50
CNE55	AE	< 50	< 50	< 50	< 50	< 50
CNE8	AE	< 50	< 50	51	< 50	< 50
X2278	B	< 50	< 50	< 50	< 50	< 50
TRO11	B	< 50	< 50	< 50	< 50	< 50
BJOX2000	BC	< 50	< 50	< 50	< 50	< 50
CH119	BC	< 50	< 50	< 50	< 50	< 50
CE0217	C	< 50	< 50	< 50	< 50	< 50
CE1176	C	< 50	< 50	< 50	< 50	< 50
25710	C	< 50	< 50	< 50	< 50	< 50
X1632	G	< 50	< 50	< 50	< 50	< 50

Supplementary Figure 5. Binding and neutralization titers for 327c immunized (A) V3 binding titers for 327c SOSIP-immunized rabbits at wk26. V3-peptides correspond to V3 sequence found in each trimer WT 327c, MD39 and MD37. (B) MW965 neutralization titers at wk26 and wk34. (C) Neutralization titers at wk34 against 12 virus panel. Measurements were done once. Statistical comparison were calculated using two-tailed Mann-Whitney U tests. * indicates $p < 0.05$, ** indicates $p < 0.01$.

```
327c SOSIP             MGNLWVTVYYGVPVWKEAKTTLFCASDAKAYEQEVHNVWATHACVPTDPNPQEMRMENVT
327c MD37              MGNLWVTVYYGVPVWKEAKTTLFCASDAKAYEQEVHNVWATHACVPTDPNPQEMRMENVT
327c MD39              MGNLWVTVYYGVPVWKEAKTTLFCASDAKAYEQEVHNVWATHACVPTDPNPQEMRMENVT
AD8 SOSIP             VENLWVTVYYGVPVWKEATTTLFCASDAKAYDTEVHNVWATHACVPTDPNPQEVVLENVT
AD8 MD64              VENLWVTVYYGVPVWKEATTTLFCASDAKAYDTEVHNVWATHECVPTDPNPQEVVLENVT
BG505 SOSIP           AENLWVTVYYGVPVWKDAETTLFCASDAKAYETEKHNVWATHACVPTDPNPQEIHLENVT
BG505 MD39            AENLWVTVYYGVPVWKDAETTLFCASDAKAYETEKHNVWATHACVPTDPNPQEIHLENVT
BG505 MD39.GRSF6      AENLWVTVYYGVPVWKDAETTLFCASDAKAYETEKHNVWATHACVPTDPNSSEIHLENVT
BG505 MD39.GRSF8      AENLWVTVYYGVPVWKDAETTLFCASDAKAYETEKHNVWATHACVPTDPNSSEIHLENVT
BG505 MD37            AENLWVTVYYGVPVWKDAETTLFCASDAKAYETEKHNVWATHACVPTDPNPQEIHLENVT
BG505 MD64            AENLWVTVYYGVPVWKDAETTLFCASDAKAYETEKHNVWATHECVPTDPNPQEIHLENVT
BG505 Olio6           AENLWVTVYYGVPVWKDAETTLFCASDAKAYETEKHNVWATHACVPTDPNPQEIHLENVT
BG505 Olio6.CD4KO     AENLWVTVYYGVPVWKDAETTLFCASDAKAYETEKHNVWATHACVPTDPNPQEIHLENVT

327c SOSIP             ENFNMWKNDMVDQMHEDIISLWDQSLKPCVKLTPLCVTLDCQNVNATQNTN----DTIST
327c MD37              ENFNMWKNDMVDQMHEDIISLWDQSLKPCCKLTPLCVTLDCQNVNATQNTN----DTIST
327c MD39              ENFNMWKNDMVDQMHEDIISLWDQSLKPCVKLTPLCVTLDCQNVNATQNTN----DTIST
AD8 SOSIP             ENFNMWKNNMVEQMHEDIISLWDQSLKPCVKLTPLCVTLNCTDLRNVTNINNSSEGMRGE
AD8 MD64              ENFNMWKNNMVEQMHEDIIELWDQSLKPCVKLTPLCVTLNCTDLRNVTNINNSSEGMRGE
BG505 SOSIP           EEFNMWKNNMVEQMHTDIISLWDQSLKPCVKLTPLCVTLQCTNVTN-----NITDDMRGE
BG505 MD39            EEFNMWKNNMVEQMHEDIISLWDQSLKPCVKLTPLCVTLQCTNVTN-----NITDDMRGE
BG505 MD39.GRSF6      EEFNMWKNNMVEQMHEDIISLWDQSLKPCVKLTPLCVTLQCTNVTN-----NITDDMRGE
BG505 MD39.GRSF8      EEFNMWKNNMVEQMHEDIISLWDQSLKPCVKLTPLCVTLQCTNVTN-----NITDDMRGE
BG505 MD37            EEFNMWKNNMVEQMHEDIISLWDQSLKPCCKLTPLCVTLQCTNVTN-----NITDDMRGE
BG505 MD64            EEFNMWKNNMVEQMHTDIIELWDQSLKPCVKLTPLCVTLQCTNVTN-----NITDDMRGE
BG505 Olio6           EEFNMWKNNMVEQMHEDIISLWDQSLKPCVKLTPLCVTLQCTNVTN-----NITDDMRGE
BG505 Olio6.CD4KO     EEFNMWKNNMVEQMHEDIISLWDQSLKPCVKLTPLCVTLQCTNVTN-----NITDDMRGE

327c SOSIP             MKNCTFNTTADLGDKKQKGRALFYNLDIVQLNPNS------NSSEYRLISCNTSTITQAC
327c MD37              MKNCTFNTTADLGDKKQKGRALFYNLDIVQLNPNS------NSSEYRLISCNTSTCTQAC
327c MD39              MKNCTFNTTADLGDKKQKGRALFYNLDIVQLNPNS------NSSEYRLISCNTSTITQAC
AD8 SOSIP             IKNCSFNITTSIRDKVKKDYALFYRLDVVPIDN--------DNTSYRLINCNTSTITQAC
AD8 MD64              IKNCSFNITTSIRDKVKKDYALFYRLDVVPIDN--------DNTSYRLINCNTSTITQAC
BG505 SOSIP           LKNCSFNMTTELRDKKQKVYSLFYRLDVVQINENQGNRSNNSNKEYRLINCNTSAITQAC
BG505 MD39            LKNCSFNMTTELRDKKQKVYSLFYRLDVVQINENQGNRSNNSNKEYRLINCNTSAITQAC
BG505 MD39.GRSF6      LKNCSFNMTTELRDKKQKVYSLFYRLDVVQINENQGNRSNNSNKEYRLINCNTSAITQAC
BG505 MD39.GRSF8      LKNCSFNMTTELRDKKQKVYSLFYRLDVVQINENQGNRSNNSNKEYRLINCNTSAITQAC
BG505 MD37            LKNCSFNMTTELRDKKQKVYSLFYRLDVVQINENQGNRSNNSNKEYRLINCNTSACTQAC
BG505 MD64            LKNCSFNMTTELRDKKQKVYSLFYRLDVVQINENQGNRSNNSNKEYRLINCNTSAITQAC
BG505 Olio6           LKNCSFNMTTELRDKKQKVYSLFWRLDVVQINENQGNRSNNSNKEYRLINCNTSAITQAC
BG505 Olio6.CD4KO     LKNCSFNMTTELRDKKQKVYSLFWRLDVVQINENQGNRSNNSNKEYRLINCNTSAITQAC

327c SOSIP             PKVSFEPIPIHYCAPAGYAILKCNNKTFNGLGPCTNVSTVQCTHGIKPVVSTQLLLNGSL
327c MD37              PKVSFEPIPIHYCAPAGYAILKCNNKTFNGLGPCTNVSTVQCTHGIKPVVSTQLLLNGSL
327c MD39              PKVSFEPIPIHYCAPAGYAILKCNNKTFNGLGPCTNVSTVQCTHGIKPVVSTQLLLNGSL
AD8 SOSIP             PKVSFEPIPIHYCTPAGFAILKCKDKKFNGTGPCKNVSTVQCTHGIRPVVSTQLLLNGSL
AD8 MD64              PKVSFEPIPIHYCTPAGFAILKCKDKKFNGTGPCKNVSTVQCTHGIRPVVSTQLLLNGSL
BG505 SOSIP           PKVSFEPIPIHYCAPAGFAILKCKDKKFNGTGPCPSVSTVQCTHGIKPVVSTQLLLNGSL
BG505 MD39            PKVSFEPIPIHYCAPAGFAILKCKDKKFNGTGPCPSVSTVQCTHGIKPVVSTQLLLNGSL
BG505 MD39.GRSF6      PKVSFEPIPIHYCAPAGFAILKCKDKKFNGTGPCQNVSTVQCTHGIKPVVSTQLLLNGSL
BG505 MD39.GRSF8      PKVSFEPIPIHYCAPAGFAILKCKDKKFNGTGPCQNVSTVQCTHGIKPVVSTQLLLNGSL
BG505 MD37            PKVSFEPIPIHYCAPAGFAILKCKDKKFNGTGPCPSVSTVQCTHGIKPVVSTQLLLNGSL
BG505 MD64            PKVSFEPIPIHYCAPAGFAILKCKDKKFNGTGPCPSVSTVQCTHGIKPVVSTQLLLNGSL
BG505 Olio6           PKVSFEPIPIHYCAPAGFAILKCKDKKFNGTGPCPSVSTVQCTHGIKPVVSTQLLLNGSL
BG505 Olio6.CD4KO     PKVSFEPIPIHYCAPAGFAILKCKDKKFNGTGPCPSVSTVQCTHGIKPVVSTQLLLNGSL

327c SOSIP             AEGEIIIRSENLTDNGKTIIVHLNESVKIVCIRPNNNTRKSIRIGPGQTFFA-TDIIGDI
327c MD37              AEGEIIIRSENLTDNGKTIIVHLNESVKIVCIRPNNNTRKSIRIGPGCTFFA-TDIIGDI
327c MD39              AEGEIIIRSENLTDNGKTIIVHLNESVKIVCIRPNNNTVKSIRIGPGQTFFY-TDIIGDI
AD8 SOSIP             AEEEVVIRSSNFTDNAKNIIVQLKESVEINCTRPNNNTRKSIHIGPGRAFYTTGDIIGDI
AD8 MD64              AEEEVIIRSSNFTDNAKNIIVQLKESVEINCTRPNNNTVKSIHIGPGRAFYYTGDIIGDI
BG505 SOSIP           AEEEVMIRSENITNNAKNILVQFNTPVQINCTRPNNNTRKSIRIGPGQAFYATGDIIGDI
BG505 MD39            AEEEVIIRSENITNNAKNILVQLNTPVQINCTRPNNNTVKSIRIGPGQAFYYTGDIIGDI
BG505 MD39.GRSF6      AEEEVIIRSENITNNAKNILVQLNTSVQINCTRPNNNTVKSIRIGPGQAFYYTGDIIGDI
BG505 MD39.GRSF8      AEEEVIIRSENITNNAKNILVQLNTSVQINCTRPNNNTVKSIRIGPGQAFYYTGDIIGDI
BG505 MD37            AEEEVIIRSENITNNAKNILVQLNTPVQINCTRPNNNTRKSIRIGPGCAFYATGDIIGDI
BG505 MD64            AEEEVIIRSENITNNAKNILVQLNTPVQINCTRPNNNTVKSIRIGPGQAFYYTGDIIGDI
BG505 Olio6           AEEEVIIRSENITNNAKNILVQLNTPVQINCTAPNNFTVKSIRIGPGQAFYYMGDIIGDI
BG505 Olio6.CD4KO     AEEEVIIRSENITNNAKNILVQLNTPVQINCTAPNNFTVKSIRIGPGQAFYYMGDIIGDI
```

```
327c SOSIP            RQAYCNISREDWNKTLDRVRKKLEEHFPN-KTIEFKRHSGGDLEVTQHSFNCRGEFFYCN
327c MD37             RQAYCNISREDWNKTLDRVRKKLEEHFPN-KTIEFKQHSGGDLEVTQHSFNCRGEFFYCN
327c MD39             RQAYCNISREDWNKTLDRVRKKLEEHFPN-KTIEFKQHSGGDLEVTQHSFNCRGEFFYCN
AD8 SOSIP             RQAHCNISRTKWNNTLNQIATKLKEQFGNNKTIVFNQSSGGDPEIVMHSFNCGGEFFYCN
AD8 MD64              RQAHCNISRTKWNNTLNQIATKLKEQFGNNKTIVFNQSSGGDPEIVMHSFNCGGEFFYCN
BG505 SOSIP           RQAHCNVSKATWNETLGKVVKQLRKHFGNNTIIRFANSSGGDLEVTTHSFNCGGEFFYCN
BG505 MD39            RQAHCNVSKATWNETLGKVVKQLRKHFGNNTIIRFAQSSGGDLEVTTHSFNCGGEFFYCN
BG505 MD39.GRSF6      RQAHCNVSKATWNETLGKVVKQLRKHFGNNTIIRFAQSSGGDLEVTTHSFNCGGEFFYCN
BG505 MD39.GRSF8      RQAHCNVSKATWNETLGKVVKQLRKHFGNNTIIRFAQSSGGDLEVTTHSFNCGGEFFYCN
BG505 MD37            RQAHCNVSKATWNETLGKVVKQLRKHFGNNTIIRFAQSSGGDLEVTTHSFNCGGEFFYCN
BG505 MD64            RQAHCNVSKATWNETLGKVVKQLRKHFGNNTIIRFAQSSGGDLEVTTHSFNCGGEFFYCN
BG505 Olio6           RQAHCNVSKATWNETLGKVVKQLRKHFGNNTIIRFAQSSGGDLEVTTHSFNCGGMFFYCN
BG505 Olio6.CD4KO     RQAHCNVSKATWNETLGKVVKQLRKHFGNNTIIRFAQSSGGDLEVTTHSFNCGGMFFYCN

327c SOSIP            TTHLFENTTYT-------------NSSNITLPCRIKQIINMWQGVGRAMYAPPIAGNITC
327c MD37             TTHLFENTTYT-------------NSSNITLPCRIKQIINMWQGVGRCMYAPPIAGNITC
327c MD39             TTHLFENTTYT-------------NSSNITLPCRIKQIINMWQGVGRAMYAPPIAGNITC
AD8 SOSIP             STQLFNSTWNFNGTWNLTQSNGTEGNDTITLPCRIKQIINMWQEVGKAMYAPPIRGQIRC
AD8 MD64              STQLFNSTWNFNGTWNLTQSNGTEGNDTITLPCRIKQIINMWQEVGKAMYAPPIRGQIRC
BG505 SOSIP           TSGLFNSTWISNTSV--QGSNSTGSNDSITLPCRIKQIINMWQRIGQAMYAPPIQGVIRC
BG505 MD39            TSGLFNSTWISNTSV--QGSNSTGSNDSITLPCRIKQIINMWQRIGQAMYAPPIQGVIRC
BG505 MD39.GRSF6      TSGLFNSTWISNTSV--QGSNSTGSNDSITLPCRIKQIINMWQRIGQAMYAPPIQGVIRC
BG505 MD39.GRSF8      TSGLFNSTWISNTSV--QGSNSTGSNDSITLPCRIKQIINMWQRIGQAMYAPPIQGVIRC
BG505 MD37            TSGLFNSTWISNTSV--QGSNSTGSNDSITLPCRIKQIINMWQRIGQCMYAPPIQGVIRC
BG505 MD64            TSGLFNSTWISNTSV--QGSNSTGSNDSITLPCRIKQIINMWQRIGQAMYAPPIQGVIRC
BG505 Olio6           TSGLFNSTWISNTSV--QGSNSTGSNDSITLPCRIKLIINMWQRIGQAMYAPPIQGVIRC
BG505 Olio6.CD4KO     TSGLFNSTWISNTSV--QGSNSTGSNDSITLPCRIKLIINMWQRIGQAMYAPPIQGVIRC

327c SOSIP            ISNITGILLTRDGGNN--GTNETFRPGGGDMRDNWRSELYKYKVVEIKPLGIAPTKCKRR
327c MD37             ISNITGILLTRDGGNN—GTNETFRPGGGDMRDNWRSELYKYKVVEIKPLGIAPTKCKRR
327c MD39             ISNITGILLTRDGGNN--GTNETFRPGGGDMRDNWRSELYKYKVVEIKPLGIAPTKCKRR
AD8 SOSIP             SSNITGLILTRDGGNNHNNDTETFRPGGGDMRDNWRSELYKYKVVKIEPLGVAPTKCKRR
AD8 MD64              SSNITGLILTRDGGNNHNNDTETFRPGGGDMRDNWRSELYKYKVVKIEPLGVAPTKCKRR
BG505 SOSIP           VSNITGLILTRDGGST-NSTTETFRPGGGDMRDNWRSELYKYKVVKIEPLGVAPTRCKRR
BG505 MD39            VSNITGLILTRDGGST-NSTTETFRPGGGDMRDNWRSELYKYKVVKIEPLGVAPTRCKRR
BG505 MD39.GRSF6      VSNITGLILTRDGGST-NSTTETFRPGGGDMRDNWRSELYKYKVVKIEPLGVAPTRCKRR
BG505 MD39.GRSF8      VSNITGLILTRDGGST-NSTTETFRPGGGDMRDNWRSELYKYKVVKIEPLGVAPTRCKRR
BG505 MD37            VSNITGLILTRDGGST-NSTTETFRPGGGDMRDNWRSELYKYKVVKIEPLGVAPTRCKRR
BG505 MD64            VSNITGLILTRDGGST-NSTTETFRPGGGDMRDNWRSELYKYKVVKIEPLGVAPTRCKRR
BG505 Olio6           VSNITGLILTRDGGST-NSTTETFRPGGGDMRDNWRSELYKYKVVKIEPLGVAPTRCKRR
BG505 Olio6.CD4KO     VSNITGLILTRDGGST-NSTTETFRPGGTDMRDNWRSELYKYKVVKIEPLGVAPTRCKRR

327c SOSIP            VVERRRRRRAVG-MGALFLGFLGAAGSTMGAASMTLTVQARQLLSGIVQQQSNLLRAPEA
327c MD37             VVERRRRRRAVG-MGALSLGFLGAAGSTMGAASMTLTVQARQLLSGIVQQQSNLLRAPEP
327c MD39             VVERRRRRRAVG-MGALSLGFLGAAGSTMGAASMTLTVQARQLLSGIVQQQSNLLRAPEP
AD8 SOSIP             VVQRRRRRRAVGTIGAMFLGFLGAAGSTMGAASITLTVQARLLLSGIVQQQNNLLRAPEA
AD8 MD64              VVQRRRRRRAVGTIGAMSLGFLGAAGSTMGAASITLTVQARLLLSGIVQQQNNLLRAPEP
BG505 SOSIP           VVGRRRRRRAVG-IGAVFLGFLGAAGSTMGAASMTLTVQARNLLSGIVQQQSNLLRAPEA
BG505 MD39            VVGRRRRRRAVG-IGAVSLGFLGAAGSTMGAASMTLTVQARNLLSGIVQQQSNLLRAPEP
BG505 MD39.GRSF6      VVGRRRRRRAVG-IGAVSLGFLGAAGSTMGAASMTLTVQARNLLSGIVQQQSNLLRAPEP
BG505 MD39.GRSF8      VVGRRRRRRAVG-IGAVSLGFLGAAGSTMGAASMTLTVQARNLLSGIVQQQSNLLRAPEP
BG505 MD37            VVGRRRRRRAVG-IGAVSLGFLGAAGSTMGAASMTLTVQARNLLSGIVQQQSNLLRAPEP
BG505 MD64            VVGRRRRRRAVG-IGAVSLGFLGAAGSTMGAASMTLTVQARNLLSGIVQQQSNLLRAPEP
BG505 Olio6           VVGRRRRRRAVG-IGAVSLGFLGAAGSTMGAASMTLTVQARNLLSGIVQQQSNLLRAPEP
BG505 Olio6.CD4KO     VVGRRRRRRAVG-IGAVSLGFLGAAGSTMGAASMTLTVQARNLLSGIVQQQSNLLRAPEP

327c SOSIP            QQHMLQLTVWGIKQLQARVLALERYLQDQQLLGIWGCSGKLICCTAVPWNSSWSNKTYND
327c MD37             QEHMHQDTHWGIKQLQARVLALEHYLQDQQLLGIWGCSGKLICCTAVPWNSSWSNKTYND
327c MD39             QQHMLQDTHWGIKQLQARVLALEHYLQDQQLLGIWGCSGKLICCTAVPWNSSWSNKTYND
AD8 SOSIP             QQHLLQLTVWGIKQLQARVLAVERYLRDQQLLGIWGCSGKLICCTAVPWNASWSNKTLDM
AD8 MD64              QQHLLQLTVWGIKQLQARVLAVEHYLRDQQLLGIWGCSGKLICCTAVPWNASWSNKTLDM
BG505 SOSIP           QQHLLKLTVWGIKQLQARVLAVERYLRDQQLLGIWGCSGKLICCTNVPWNSSWSNRNLSE
BG505 MD39            QQHLLKDTHWGIKQLQARVLAVEHYLRDQQLLGIWGCSGKLICCTNVPWNSSWSNRNLSE
BG505 MD39.GRSF6      QQHLLKDTHWGIKQLQARVLAVEHYLRDQQLLGIWGCSGKLICCTNVPWNSSWSNANLSE
BG505 MD39.GRSF8      QQHLLKDTHWGIKQLQARVLAVEHYLRDQQLLGIWGCSGKLICCTNVPWNSSWSNRNLSE
BG505 MD37            QEHLHKDTHWGIKQLQARVLAVEHYLRDQQLLGIWGCSGKLICCTNVPWNSSWSNRNLSE
BG505 MD64            QQHLLKLTVWGIKQLQARVLAVEHYLRDQQLLGIWGCSGKLICCTNVPWNSSWSNRNLSE
BG505 Olio6           QQHLLKDTHWGIKQLQARVLAVEHYLRDQQLLGIWGCSGKLICCTNVPWNSSWSNRNLSE
BG505 Olio6.CD4KO     QQHLLKDTHWGIKQLQARVLAVEHYLRDQQLLGIWGCSGKLICCTNVPWNSSWSNRNLSE
```

```
327c SOSIP            IWDNMTWMQWEREISNYTNTIYTLLEVSQNQQEQNEKDLLALD
327c MD37             IWDNMTWMQWEREISNYTNTIYTLLEVSQNQQEQNEKDLLALD
327c MD39             IWDNMTWMQWEREISNYTNTIYTLLEVSQNQQEQNEKDLLALD
AD8 SOSIP             IWNNMTWMEWEREIDNYTGLIYTLIEESQNQQEKNEQELLELD
AD8 MD64              IWNNMTWMEWEREIDNYTGLIYTLIEESQNQQEKNEQELLELD
BG505 SOSIP           IWDNMTWLQWDKEISNYTQIIYGLLEESQNQQEKNEQDLLALD
BG505 MD39            IWDNMTWLQWDKEISNYTQIIYGLLEESQNQQEKNEQDLLALD
BG505 MD39.GRSF6      IWDNMTWLQWDKEISNYTQIIYGLLEESQNQNESNEQDLLALDNGS
BG505 MD39.GRSF8      IWDNMTWLNWSKEISNYTQIIYGLLEESQNQQEKNNQSLLALDNGS
BG505 MD37            IWDNMTWLQWDKEISNYTQIIYGLLEESQNQQEKNEQDLLALD
BG505 MD64            IWDNMTWLQWDKEISNYTQIIYGLLEESQNQQEKNEQDLLALD
BG505 Olio6           IWDNMTWLQWDKEISNYTQIIYGLLEESQNQQEKNEQDLLALD
BG505 Olio6.CD4KO     IWDNMTWLQWDKEISNYTQIIYGLLEESQNQQEKNEQDLLALD
```

Supplementary Figure 6. Sequences of native-like trimers. The sequons for new n-linked glycans are highlighted yellow. For 327c and AD8 trimers, mutations from the base SOSIP are highlighted green. For BG505 trimers, newly engineered mutations reported in this manuscript are highlighted green.

	GP41
BG505_MD39.CP	-----VSLGFLGAAGSTMGAASMTLTVQARNLLSGIVQQQSNLLRAPEPQQHLLKDTHWG
BG505_MD39.CP.GRSF4	-----VSLGFLGAAGSTMGAASMTLTVQARNLLSGIVQQQSNLLRAPEPQQHLLKDTHWG
BG505_MD39.CP.GRSF9	GGNSSGSLGFLGAAGSTMGAASMTLTVQARNLLSGIVQQQSNLLRAPEPQQHLLKDTHWG

	GP41
BG505_MD39.CP	IKQLQARVLAVEHYLRDQQLLGIWGCSGKLICCTNVPWNSSWSNRNLSEIWDNMTWLQWD
BG505_MD39.CP.GRSF4	IKQLQARVLAVEHYLRDQQLLGIWGCSGKLICCTNVPWNSSWSNRNLSEIWDNMTWLQWD
BG505_MD39.CP.GRSF9	IKQLQARVLAVEHYLRDQQLLGIWGCSGKLICCTNVPWNSSWSNRNLSEIWDNMTWLNWS

	GP41 GP120
BG505_MD39.CP	KEISNYTQIIYGLLEESQNQQEKNEQDL**GGSGSGGGSGSGGSSGSG**LWVTVYYGVPVWKD
BG505_MD39.CP.GRSF4	KEISNYTQIIYGLLEESQNQQEKNEQDL**GGNGSGGGSGSGGNGSSG**LWVTVYYGVPVWKD
BG505_MD39.CP.GRSF9	KEISNYTQIIYGLLEESQNQNESNEQDL**GGNGSGGGSGSGGNGSSG**LWVTVYYGVPVWKD

	GP120
BG505_MD39.CP	AETTLFCASDAKAYETEKHNVWATHACVPTDPNPQEIHLENVTEEFNMWKNNMVEQMHED
BG505_MD39.CP.GRSF4	AETTLFCASDAKAYETEKHNVWATHACVPTDPNSSEIHLENVTEEFNMWKNNMVEQMHED
BG505_MD39.CP.GRSF9	AETTLFCASDAKAYETEKHNVWATHACVPTDPNSSEIHLENVTEEFNMWKNNMVEQMHED

	GP120
BG505_MD39.CP	IISLWDQSLKPCVKLTPLCVTLQCTNVTNNITDDMRGELKNCSFNMTTELRDKKQKVYSL
BG505_MD39.CP.GRSF4	IISLWDQSLKPCVKLTPLCVTLQCTNVTNNITDDMRGELKNCSFNMTTELRDKKQKVYSL
BG505_MD39.CP.GRSF9	IISLWDQSLKPCVKLTPLCVTLQCTNVTNNITDDMRGELKNCSFNMTTELRDKKQKVYSL

	GP120
BG505_MD39.CP	FYRLDVVQINENQGNRSNNSNKEYRLINCNTSAITQACPKVSFEPIPIHYCAPAGFAILK
BG505_MD39.CP.GRSF4	FYRLDVVQINENQGNRSNNSNKEYRLINCNTSAITQACPKVSFEPIPIHYCAPAGFAILK
BG505_MD39.CP.GRSF9	FYRLDVVQINENQGNRSNNSNKEYRLINCNTSAITQACPKVSFEPIPIHYCAPAGFAILK

	GP120
BG505_MD39.CP	CKDKKFNGTGPCPSVSTVQCTHGIKPVVSTQLLLNGSLAEEEVIIRSENITNNAKNILVQ
BG505_MD39.CP.GRSF4	CKDKKFNGTGPCQNVSTVQCTHGIKPVVSTQLLLNGSLAEEEVIIRSENITNNAKNILVQ
BG505_MD39.CP.GRSF9	CKDKKFNGTGPCQNVSTVQCTHGIKPVVSTQLLLNGSLAEEEVIIRSENITNNAKNILVQ

	GP120
BG505_MD39.CP	LNTPVQINCTRPNNNTVKSIRIGPGQAFYYTGDIIGDIRQAHCNVSKATWNETLGKVVKQ
BG505_MD39.CP.GRSF4	LNTSVQINCTRPNNNTVKSIRIGPGQAFYYTGDIIGDIRQAHCNVSKATWNETLGKVVKQ
BG505_MD39.CP.GRSF9	LNTSVQINCTRPNNNTVKSIRIGPGQAFYYTGDIIGDIRQAHCNVSKATWNETLGKVVKQ

	GP120
BG505_MD39.CP	LRKHFGNNTIIRFAQSSGGDLEVTTHSFNCGGEFFYCNTSGLFNSTWISNTSVQGSNSTG
BG505_MD39.CP.GRSF4	LRKHFGNNTIIRFAQSSGGDLEVTTHSFNCGGEFFYCNTSGLFNSTWISNTSVQGSNSTG
BG505_MD39.CP.GRSF9	LRKHFGNNTIIRFAQSSGGDLEVTTHSFNCGGEFFYCNTSGLFNSTWISNTSVQGSNSTG

	GP120
BG505_MD39.CP	SNDSITLPCRIKQIINMWQRIGQAMYAPPIQGVIRCVSNITGLILTRDGGSTNSTTETFR
BG505_MD39.CP.GRSF4	SNDSITLPCRIKQIINMWQRIGQAMYAPPIQGVIRCVSNITGLILTRDGGSTNSTTETFR
BG505_MD39.CP.GRSF9	SNDSITLPCRIKQIINMWQRIGQAMYAPPIQGVIRCVSNITGLILTRDGGSTNSTTETFR

	GP120
BG505_MD39.CP	PGGGDMRDNWRSELYKYKVVKIEPLGVAPTRCKRR
BG505_MD39.CP.GRSF4	PGGGDMRDNWRSELYKYKVVKIEPLGVAPTRCKRR
BG505_MD39.CP.GRSF9	PGGGDMRDNWRSELYKYKVVKIEPLGVAPTRCNRS

Supplementary Figure 7. Sequences of CP native-like trimers. The sequons for new n-linked glycans are highlighted in yellow. The linker sequence between gp41 c-terminus and gp120 n-terminus is bolded.

			V1V2 apex		N332 supersite		CD4 binding site		gp120-gp41 interface		
Strain	Stabilization	Clade	PGDM 1400	PGT 145	PGT 121	PGT 128	12A12	3BNC60	PGT 151	TM (°C)	Yield
BG505	SOSIP.664	A	2	6	22	17	97	137	10	66	1
	MD37	A		17		19	106	189		80	1
	MD39	A	4	11	21	8	67	121	11	77	6.5
	Olio6	A	6	8	15	11	40	119		76	2.3
	MD64	A		23		14	71	144	15	82.5	5.4
191084	MD39	A1			26	25	77	178			1.7
AC10	MD39	B	63	21	169	33	277			60	8.6
AD8	MD39	B		37	23	29	184	91		61	1.5
	MD64	B		38	20	18	134	54		67	1.1
SF162P3	MD64	B	320		12	14	98	100			4.2
001428	MD39	C			16	42	55	74			4.6
327c	SOSIP.664	C	38	114	118	WB	359	451		60/65	1.5
	MD37	C	94	178	90	WB	523	329		69	1.8
	MD39	C	142	159	40	130	416	208		61	4

Supplementary Table 1. Biophysical properties of stabilized variants of native-like trimers. The Dissociation constants (K_Ds) for bnAbs were measured by SPR and are reported in units of nM as described in Figure 1. The melting temperatures were measured by DSC, and the yields were determined by comparing amounts of protein purified from 293F-transfected cells. WB, weak binding. Data are from one or more experiments.
